# The Pseudokinase TRIB3 Negatively Regulates the HER2 Receptor Pathway and Is a Biomarker of Good Prognosis in Luminal Breast Cancer

**DOI:** 10.3390/cancers13215307

**Published:** 2021-10-22

**Authors:** Alba Orea-Soufi, Sonia Castillo-Lluva, Nélida Salvador-Tormo, Paola Martín-Cabrera, Silvia Recuero, Estíbaliz Gabicagogeascoa, Manuel Moreno-Valladares, Marina Mendiburu-Eliçabe, Adrián Blanco-Gómez, José Miguel Ramos-Pittol, Elena García-Taboada, Alberto Ocaña, Francisco J. Cimas, Ander Matheu, Isabel Álvarez-López, Guillermo Velasco, Mar Lorente

**Affiliations:** 1 Department of Biochemistry and Molecular Biology, School of Biology, Complutense University, 28040 Madrid, Spain; albaorea@ucm.es (A.O.-S.); sonica01@ucm.es (S.C.-L.); nsalvado@ucm.es (N.S.-T.); paolmart@ucm.es (P.M.-C.); silviarda97@gmail.com (S.R.); egabicag@ucm.es (E.G.); marinamendiburu@usal.es (M.M.-E.); elenagtaboada@gmail.com (E.G.-T.); 2 Instituto de Investigaciones Sanitarias San Carlos (IdISSC), 28040 Madrid, Spain; 3 Osakidetza Basque Health Service, Donostia University Hospital, 20014 San Sebastian, Spain; MANUEL.MORENOVALLADARES@osakidetza.eus (M.M.-V.); ISABELMANUELA.ALVAREZLOPEZ@osakidetza.eus (I.Á.-L.); 4 Department of Oncology, Biodonostia Health Research Institute, 20014 San Sebastián, Spain; ander.matheu@biodonostia.org; 5 Instituto de Biología Molecular y Celular del Cáncer (IBMCC-CIC), Universidad de Salamanca/CSIC, 37007 Salamanca, Spain; adrian.blancogomez@cruk.manchester.ac.uk; 6 Institute of Biochemistry and Center for Molecular Biosciences Innsbruck, University of Innsbruck, Innsbruck 6020, Austria; Jose.Ramos-Pittol@uibk.ac.at; 7 Experimental Therapeutics Unit, Hospital Clínico San Carlos (HCSC), Instituto de Investigaciones Sanitarias San Carlos (IdISSC), 28040 Madrid, Spain; alberto.ocana@salud.madrid.org; 8 Translational Research Unit, Translational Oncology Laboratory, Albacete University Hospital, Universidad de Castilla La Mancha (UCLM), 13071 Albacete, Spain; franciscojose.cimas@uclm.es; 9 Centro Regional de Investigaciones Biomédicas, Castilla-La Mancha University (CRIB-UCLM), 13071 Albacete, Spain; 10 IKERBASQUE, Basque Foundation for Science, 48009 Bilbao, Spain; 11 CIBER of Frailty and Healthy Aging (CIBERfes), Carlos III Institute, 28029 Madrid, Spain

**Keywords:** TRIB3, Luminal breast cancer, HER2, AKT, tissue microarrays, cell signaling

## Abstract

**Simple Summary:**

Breast cancer is the most frequent type of cancer in women. More than 70% of these tumors belong to the so-called luminal subtype which has, in general, a good prognosis. However, a fraction of patients with luminal breast cancer progress to an advanced or metastatic disease, which remains a major clinical and social problem. Therefore, it is crucial to identify novel biomarkers that help to predict the progression of the disease and to develop more efficacious therapeutic approaches to fight advanced luminal breast cancer. In this work we found that the increased expression of the protein tribbles pseudokinase 3 (TRIB3) is associated with a good prognosis and a better response to therapy in luminal breast cancer patients. We also found that this effect is at least in part due to the ability of TRIB3 to inhibit the activity of the oncogene HER2.

**Abstract:**

Background: Tribbles pseudokinase 3 (TRIB3) has been proposed to both promote and restrict cancer generation and progression. However, the precise mechanisms that determine this dual role of TRIB3 in cancer remain to be understood. In this study we aimed to investigate the role of TRIB3 in luminal breast cancer, the most frequent subtype of this malignancy. Methods: We genetically manipulated TRIB3 expression in a panel of luminal breast cancer cell lines and analyzed its impact on cell proliferation, and the phosphorylation, levels, or subcellular localization of TRIB3 and other protein regulators of key signaling pathways in luminal breast cancer. We also analyzed TRIB3 protein expression in samples from luminal breast cancer patients and performed bioinformatic analyses in public datasets. Results: TRIB3 enhanced the proliferation and AKT phosphorylation in luminal A (HER2-) but decreased them in luminal B (HER2+) breast cancer cell lines. TRIB3 negatively regulated the stability of HER2 in luminal B breast cancer cell lines. TRIB3 expression was associated with increased disease-free survival and a better response to therapy in luminal breast cancer patients. Conclusions: Our findings support the exploration of TRIB3 as a potential biomarker and therapeutic target in luminal breast cancer.

## 1. Introduction

Breast cancer (BC) is the most common malignancy in women [1]. Remarkably, although breast cancer patients’ survival has increased significantly in the last two decades, mostly due to early detection and the implementation of targeted therapies, this type of tumor remains the main cause of cancer-related death in women in most countries [2]. Breast cancer is a highly heterogeneous disease where patients present different clinical, histopathological, and molecular features. Thus, one of the breakthroughs in cancer treatment has been the molecular stratification of breast cancer patients which has facilitated the development of specific treatments for each cancer subtype. The standard diagnosis of breast tumors is currently based on their histopathological characterization together with different molecular biomarkers. Specifically, the immunohistochemical analysis of estrogen receptors (ER), progesterone receptors (PR), and the human epidermal growth factor receptor 2 (HER2) remains the standard procedure to stratify breast cancer patients [3,4]. There are at least five distinct molecular breast cancer subtypes: luminal A (ER+/PR+/HER2−/low Ki-67 low proliferation rate); luminal B (ER+/PR+/HER2−/+/high Ki-67 high proliferation rate); HER2-enriched (ER−/PR−/HER2+); and triple negative breast cancers/TNBCs (ER−/PR−/HER2− and positive for typical basal cell cytokines) [5].

Approximately 75% of breast cancers are luminal (positive for ER, PR, or both and expressing markers of luminal epithelial cells). Within luminal tumors, the most common subtype is luminal A which comprises 50–60% of all diagnosed breast cancers. These luminal A tumors are frequently low-grade and HER2− [6] and their treatment is mainly based on endocrine therapy (the blockade of ER receptors or the inhibition of the aromatase enzyme) [7]. Luminal B tumors represent 15–20% of breast cancers. In approximately one third of these tumors HER2 is overexpressed [8,9]. Luminal B tumors have a more aggressive phenotype, a higher histological grade, and a worse prognosis than those of the luminal A subtype [8,9]. Patients that are overexpressing HER2 are frequently treated with Trastuzumab (an humanized monoclonal antibody against this receptor) or, in most cases upon recurrence, lapatinib (an small molecular inhibitor of HER2 tyrosine kinase activity) [10]. Despite the implementation of these selective therapies against luminal and HER2 positive breast cancers, approximately 20–30% of the patients that are treated with endocrine therapy and 70% of patients that are treated with Trastuzumab develop resistance, and progress to a more advanced disease [11]. It is, therefore, essential to identify novel biomarkers that help to predict the acquisition of resistance to these therapies and to anticipate the progression of the disease to improve the management of patients with invasive luminal breast cancer.

Tribbles pseudokinase 3 (TRIB3) is one of the three human orthologs of the *Drosophila* protein tribbles that controls cell proliferation and migration during development [12,13]. Mammalian tribbles proteins participate in the regulation of a multitude of processes, including cell cycle progression, proliferation, differentiation, stress response, cell signaling, metabolism, inflammation, immunity, and development [14,15]. These effects of tribbles proteins rely on their ability to modulate several important signaling pathways including the mitogen activated protein kinase (MAPK) cascades and the phosphatidylinositol 3 kinase (PI3K)/AKT axis [14,15]. Importantly, dysregulation of TRIB3 has been associated with different pathological conditions including cancer development [14,15,16,17]. Intriguingly, different studies have found that TRIB3 can exert both oncogenic [18,19,20,21,22,23,24] and onco-suppressive [16,25,26,27,28,29,30] actions in different cancer types, which suggest that the impact of TRIB3 dysregulation in tumorigenesis might be determined by the cellular context.

The existing literature on the role of TRIB3 in breast cancer is limited and relatively contradictory. For example, high mRNA levels of TRIB3 were associated with a poor outcome [31] while increased protein levels of TRIB3 correlated with good prognosis [32] in the same cohort of breast cancer patients. Studies that were performed in triple negative breast cancer models indicated that TRIB3 can promote tumor development and resistance to therapy [33,34]. In contrast, previous analyses have demonstrated that the TRIB3-mediated inhibition of the protein kinase AKT inhibited tumor growth in different tumor models, including breast cancer cell lines [29]. However, the role of TRIB3 in luminal breast cancer, the most frequent BC subtype [35] which often harbors alterations in the PI3K/AKT pathway [36], remains to be explored.

In the present work we found that TRIB3 differentially regulates the proliferation and the activation of the PI3K/AKT pathway of breast cancer cell lines depending on their HER2 status. In addition, we show that in HER2+ luminal B cells, TRIB3 negatively regulates HER2 signaling and associated proliferation. Importantly, analysis of samples from luminal breast cancer patients identified TRIB3 as a potential biomarker of good prognosis and response to therapy in this breast cancer subtype.

## 2. Materials and Methods

### 2.1. Cell Cultures and Plasmid Transduction

Human breast cancer cell lines MDA-MB-361, AU565, BT474, MCF7, T47D, and ZR75-B were purchased from the American Type Culture Collection. Extracts from other cell lines were kindly provided by Sonia Castillo and Cristina Sánchez (Department of Biochemistry and Molecular Biology, Complutense University). The cells were cultured in RPMI (AU565, BT474, T47D, and ZR75-B), MEM (MCF7), or DMEM (MDA-MB-361) supplemented with 10% FBS and 1% penicillin/streptomycin (all) as well as with 10 μg/mL insulin (BT474 and MCF7) and maintained at 37 °C in an atmosphere of 5% CO_2_. All of the cells were routinely tested for mycoplasma contamination. The cells were periodically monitored to confirm that they conserved their original properties and were used for no more than 35 passages to limit the acquisition of additional alterations.

Expression vectors were transiently transfected with polyethylenimine (PEI) (Sigma-Aldrich, St. Louis, MO, USA). Transient genetic knock-down was performed by selective siRNA (SMARTpool, Dharmacon) transfection with DharmaFECT 1 transfection reagent (Dharmacon). Infection with TRIB3 shRNA human lentiviral particles was performed using a pool of concentrated transduction-ready viral particles containing three target specific shRNAs (or three non-targeted control shRNA) constructs (Santa Cruz Biotechnology). After the infection of human cancer cell lines, stably silenced (or control shRNA transduced) cells were selected by incubation with puromycin.

### 2.2. Cell Viability Assays

The cells were seeded at a density of 5000/cm^2^ in their standard media. After 24 h, they were transfected with a plasmid encoding myr-AKT (a constitutively active form of this kinase) or the corresponding empty vector (kindly provided by Dr Dario Alessi, Dundee University) or serum-starved for 4 h prior to the treatment with AKT inhibitor X (Sigma-Aldrich, St. Louis, MO, USA) or lapatinib (Sigma-Aldrich, St. Louis, MO, USA) or the corresponding vehicle. The cell cultures were treated with the different drugs for 24 h or 48 h, fixed and stained with crystal violet solution (0.1% crystal violet, 20% methanol in H_2_O) for 30 min. After intensive washing with water, the stained cells were solubilized in 0.12% Triton x-100 for 30 min and absorbance was measured at 570 nm. Unless otherwise indicated, the drugs were prepared in DMSO for in vitro experiments. Control incubations contained the same amount of DMSO and no significant effect was observed in any of the parameters determined throughout this study at the final concentration used (<0.5%, *v*/*v*).

### 2.3. RT-PCR Analysis

RNA was isolated using NucleoSpin RNA (Macherey-Nagel, Düren, Germany) following the manufacturer’s instructions. cDNA was subsequently obtained using a Transcriptor First Strand cDNA Synthesis Kit (Roche Applied Science, Penzberg, Germany). PCR quantitative reactions were performed using FastStart Universal SYBR (Roche, Penzberg, Germany). Human TRIB3 sense (5′-GCCACTGCCTCCCGTCTTG-3′) and antisense (5′-GCTGCCTTGCCCGAGTATGA-3′) primers were used to determine TRIB3 levels and GAPDH sense (5′-GGGAAGCTCACTGGCATGGCCTTCC-3′) and antisense (5′-CATGTGGGCCATGAGGTCCACCAC-3′) primers were used to normalize as a control. The amplifications were run in a 7900 HT-Fast Real-Time PCR System (Applied Biosystems, Waltham, MA, USA).

### 2.4. Western Blot

Western blot analysis was performed following standard procedures. Briefly, proteins were extracted using RIPA buffer [150 mM NaCl, 1% (*v*/*v*) NP40, 50 mM Tris-HCl pH 8.0, 0.1% (*v*/*v*) SDS, 1 mM EDTA, 0.5% (*w*/*v*) deoxycholate]. The total protein was quantified by using the Bradford method, resolved by SDS-PAGE on 7.5% or 10% acrylamide gels (Bio-Rad, Hercules, CA, USA) and transferred to polyvinylidene difluoride membranes. The membranes were then probed with the following primary antibodies: anti-HER2 (1:1000; Cell signaling, #2248, Danvers, MA, USA), anti-TRIB3 (1:1000; Abcam, #ab75846, Cambridge, UK), anti-pAKT S473 (1:1000; Cell signaling, #9271, Danvers, MA, USA), anti-pAKT T308 (1:1000; Cell signalling #9275, Danvers, MA, USA), anti-AKT (1:1000; Cell signaling, #9272, Danvers, MA, USA), anti-actin (1:4000; Sigma; A5441, St. Louis, MO, USA), anti-tubulin (1:4000; Sigma; T9026, St. Louis, MO, USA), or anti-HSP90 (1:2000; Sigma; SAB4200812, St. Louis, MO, USA). Antibody binding was detected with horseradish peroxidase (HRP)-conjugated anti-mouse or anti-rabbit secondary antibodies (1:5000; GE Healthcare, Chicago, IL, USA) and visualized by enhanced chemiluminescence (Bio-Rad, Hercules, CA, USA). The images were obtained with the ImageQuant LAS 500 chemiluminescence CCD camera (GE Healthcare Life Sciences, Chicago, IL, USA).

### 2.5. Cytosolic and Nuclear Fractionation

The nuclear and cytosolic fractions were obtained from total cell lysates. First, the cells were homogenised in HEPES 10 mM pH 7.9, KCl 10 mM, MgCl_2_ 1.5 mM, PMSF 1 mM, protease inhibitors 1× (Roche, Penzberg, Germany) and centrifuged at 2000× *g* during 15 min at 4 °C. The supernatant contained the cytosolic fraction. The nuclear pellet was incubated 10 min with RIPA buffer, passed through a 25 G syringe 10 times, and centrifuged for 15 min at 200× *g* at 4 °C to obtain the nuclear fraction in the supernatant. The samples were subsequently analysed by Western blot.

### 2.6. Immunostaining and Microscopy

The cells were seeded, subjected to the corresponding treatments, fixed in p-formaldehyde (PFA) for 10 min at room temperature, permeabilized, blocked with 10% goat serum in 0.25% Triton X-100 PBS for 1 h, and incubated with the primary antibody overnight at 4 °C (Ki67 1:300, Thermo Scientific #9106, Waltham, MA, USA). The cells were subsequently washed with PBS and incubated with the anti-rabbit Alexa Fluor 594 secondary antibody (Invitrogen, Waltham, MA, USA) at 1:500 dilution for 1 h at room temperature. Nuclei were stained with DAPI for 10 min (Roche, Penzberg, Germany). Finally, samples were mounted with Mowiol (Calbiochem) and the images were obtained in a ZAISS-Axioplan 2 fluoresce microscope with Metamorph-Offline 6.2 software.

### 2.7. Immunoprecipitation

The cells were lysed using a buffer containing 50 mM Tris-HCl pH 7.5, 150 mM NaCl, 0.5 M EDTA, 10 mM sodium glycerophosphate, 10 mM sodium pyrophosphate, 10% glycerol, 1% NP-40, 0.5 mM PMSF, 5 mM NaF, 1 mM Na_2_MoO_4_, 0.5 mM NaVO_4_, and protease inhibitor cocktail (Roche). The cell lysates (1–2 mg) were precleared by incubation with 10 μL of protein G–sepharose (GE Healthcare Dharmacon, Chicago, IL, USA) at 4 °C for 30 min. The lysate extracts were then incubated overnight at 4 °C with 5–10 μL of protein G–sepharose that was covalently coupled to 1.2 μg of the primary anti-TRIB3 (Abcam, ref. #75946, Cambridge, UK) or an unspecific IgG (negative control) using dimethyl pimelimidate (Sigma-Aldrich, St. Louis, MO, USA). The immunoprecipitates were washed 3 times with HEPES lysis buffer followed by 1 wash with HEPES kinase buffer (25 mM HEPES pH 7.5 and 50 mM KCl) and resuspended in 20 μL of sample buffer. The samples were subjected to electrophoresis and immunoblot analysis following standard procedures.

### 2.8. Tissue Microarrays (TMAs)

PFA-fixed and paraffin-embedded blocks of tumor tissue that were from cases operated in the University Hospital of Donostia and the Oncological Institute Onkologikoa were used for TMA construction. We obtained tumor and linfatic node samples of 291 patients. The clinical information was available for the patients, including treatment, date of local and/or distant relapse, and death. Written consent was obtained from patients for the inclusion of their samples, which were collected in accordance with the Declaration of Helsinki and approved by local ethics committees (Comité Ético de Investigación Clínica de Euskadi (CEIC-E)).

### 2.9. Immunohistological Analysis

The tissue sections were subjected to a heat-induced antigen retrieval step prior to exposure to an anti-TRIB3 antibody (Abcam, Cambridge, UK) and then followed a standard procedure. TRIB3 staining was quantified according to the intensity where 0 (no staining), 1 (weak staining), 2 (moderate staining), or 3 (high staining), and the percentage of positive cells. Next, the H value was calculated following the formula *H* = Σ (i*%*i*), *i* being each of the assigned values (0–3) and %*i* the percentage of marked cells for each of them.

### 2.10. Statistical Analyses

The data are shown as mean ± standard error of the mean (SEM). Statistical analyses were performed with GraphPad Prism 6.0 software using Student´s test, Wilcoxon test or ANOVA (One-way and Two-way ANOVA). The survival curves were analyzed by Kaplan–Meier curves and differences were compared by log-rank test analysis. A *p*-value of <0.05 was considered significant.

## 3. Results

### 3.1. TRIB3 Plays an Opposite Role in the Proliferation of Luminal A and B Breast Cancer Cell Lines

**Figure 1:**
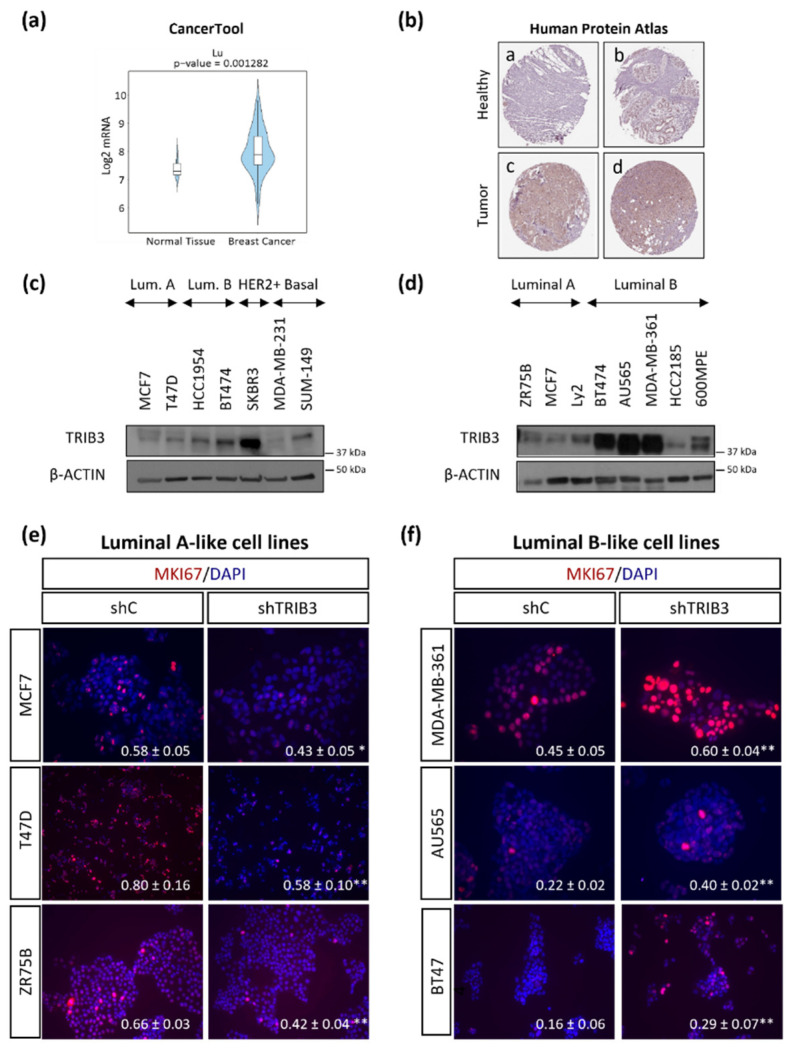
The differential role of TRIB3 in the regulation of luminal A and luminal B breast cancer cell lines: (**a**) Bioinformatic comparison of TRIB3 mRNA levels in published studies from breast cancer and healthy breast tissue (Cancertool platform; *n* = 131). (**b**) Analysis of TRIB3 protein expression (as determined by immunostaining) in healthy (**a**,**b**: not detected) and tumoral (**c**,**d**: low-intermediate levels) breast tissue. Images (40 ×) were obtained from the Human Protein Atlas database (SIGMA HPA055442 antibody). Representative images are shown. [The human protein atlas indicates that TRIB3 expression in healthy tissue is “not detectable” whereas the expression is “low to intermediate” in breast cancer tissue] (**c**,**d**) TRIB3 protein levels (as determined by Western blot) in a panel of cell lines that were representative of the different breast cancer subtypes (panel c) or of luminal breast cancer. (**e**,**f**) Effect of TRIB3 silencing on the proliferation (as determined by immunofluorescence staining with MKI67, red) of luminal A (**e**) and luminal B (**f**) breast cancer cell lines. Representative images (40 ×) are shown. Nuclei were stained with DAPI (blue). The value in the lower right corner of each microphotograph corresponds to the mean fraction ± SEM of the number of MKI67 positive cells with respect to the total number of cells. A total of ten fields per experimental condition were counted to carry out the quantifications (*n* = 4–5; * *p* < 0.05 and ** *p* < 0.01 with respect to shC cells; using the T-test).

As a first approach to investigate the role of TRIB3 in breast cancer, we analyzed the expression of this pseudokinase in public databases. We found that both TRIB3 mRNA and protein levels were upregulated in tumor samples when compared with normal breast tissue [37] (Figure 1a,b). We also investigated the relationship between TRIB3 mRNA levels and disease-free survival (DFS) in breast cancer patients using the Cancertool platform [38] and found that although several studies revealed a correlation between high TRIB3 mRNA levels and worse prognosis (Supplementary Figure S1a–d) results were variable [37,39,40,41]. Moreover, previous analyses had shown that TRIB3 mRNA and protein levels do not exhibit a clear direct correlation [31,32]. These observations prompted us to perform a more detailed characterization of the role of TRIB3 in BC. Therefore, we analyzed TRIB3 protein expression in a panel of BC cell lines that were representative of the different BC subtypes. As shown in Figure 1c,d there were striking differences in TRIB3 expression among the different cell lines (Figure 1c, the original Western blots figures can be found in File S1) and specifically between cells that were representative of the luminal A and luminal B, the most prevalent breast cancer subtypes (Figure 1d). Thus, luminal B BC cell lines (except HCC2185) exhibited higher levels of TRIB3 than luminal A BC cell lines. To further explore the role of TRIB3 in this BC subtype we genetically modified the expression of this pseudokinase in selected luminal A and B BC cell lines (Supplementary Figure S2a,b). In these analyses we found that the selective silencing of TRIB3 decreased the proliferation of MCF7, T47D, and ZR75B cell lines (that express basal low levels of TRIB3 and are considered representative of the luminal A subtype) (Figure 1e). Conversely TRIB3 overexpression increased the proliferation of MCF7 and T47D cell lines (Supplementary Figure S2c). In contrast, knock-down of TRIB3 increased the proliferation of BT474, AU565, and MDA-MB-361 cells (that express high levels of TRIB3 and are considered representative of the luminal B BC subtype; Figure 1f). Supporting the anti-proliferative role of TRIB3 in luminal B BC, further overexpression of TRIB3 on the luminal B representative cell line B7474, reduced the percentage of Ki67-positive cells (Supplementary Figure S2c). These observations pointed to the existence of a differential role of TRIB3 in the regulation of luminal A and luminal B BC cell lines proliferation.

### 3.2. TRIB3 Regulates Differently the AKT Pathway in Luminal A and B BC Cell Lines

**Figure 2:**
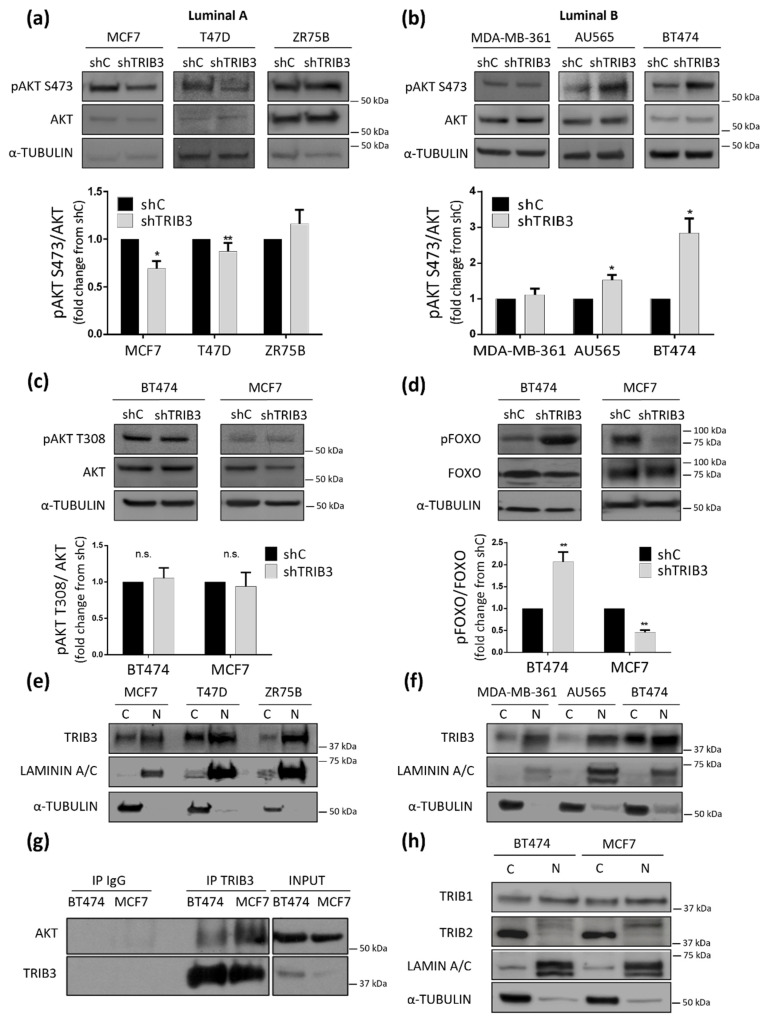
TRIB3 regulates differently the AKT pathway in luminal A and luminal B breast cancer cell lines. (**a**,**b**) The effect of TRIB3 silencing on AKT phosphorylation at serine 473 of luminal A (**a**) and luminal B (**b**) breast cancer cell lines. Upper panel: The Western blot images of a representative experiment are shown. Lower panel: The densitometric analysis of AKT phosphorylation at serine 473 (pAKT S473). The data correspond to the optical density values in arbitrary units for each experimental condition normalized with respect to the total AKT and B-ACTIN levels and are expressed as the mean fold change ± SEM with respect to shC for each case (*n* = 6–12; * *p* < 0.05, ** *p* < 0.01 with respect to shC; Wilcoxon test). (**c**) The effect of TRIB3 silencing on AKT phosphorylation at threonine 308 in BT474 (luminal B) and MCF7 (luminal A) breast cancer cell lines. Upper panel: The Western blot images of representative experiments are shown. Lower panel: The densitometric analysis of AKT phosphorylation at threonine 308 (pAKT T308). The data correspond to the optical density values in arbitrary units for each experimental condition normalized with respect to the total AKT and B-ACTIN levels and are expressed as the mean fold change ± SEM with respect to the corresponding shC condition (*n* = 6). (**d**) The effect of TRIB3 silencing on FOXO1/3 phosphorylation (FOXO1 at threonine 24 and FOXO3 at threonine 32; pFOXO1 T24/pFOXO3A T32) in BT474 (luminal B) and MCF7 (luminal A) breast cancer cell lines. Upper panel: The Western blot images of a representative experiment are shown. Lower panel: The densitometric analyses of FOXO1/3 phosphorylation. The data correspond to the optical density values in arbitrary units for each experimental condition normalized with respect to the total FOXO and B-ACTIN levels and are expressed as the mean fold change ± SEM with respect to the corresponding shC condition (*n* = 9; ** *p* < 0.01 with respect to the shC condition; using the Wilcoxon test). (**e**,**f**) TRIB3 subcellular distribution in the nuclear (N) and cytosolic (C) fractions of luminal A (**e**) and luminal B (**f**) breast cancer cells. A Western blot analysis of a representative experiment is shown. (*n* = 3; LAMININ A/C and TUBULIN are used as controls of the nuclear and cytosolic factions, respectively). (**g**) Analysis of TRIB3 and AKT coimmunoprecipitation in MCF7 and BT474 cells. TRIB3 was immunoprecipitated from lysates extracted from BT474 and MCF7 cells. IgG immunoprecipitation was included as negative control. The amount of TRIB3 and AKT was determined by Western Blot, normalizing by the total amount of protein in the lysates (input). A representative experiment is shown (*n* = 3). (**h**) The TRIB1 and TRIB2 subcellular distribution in the nuclear (N) and cytosolic (C) fractions of MCF7 and BT474 cells. A Western blot analysis of a representative experiment is shown. Note that TRIB1 is equally distributed between the nuclear and cytosolic fractions while TRIB2 is mainly located in the nuclear fraction of BT474 and MCF7 breast cancer cells. (*n* = 3; LAMININ A/C and TUBULIN are used as controls of the nuclear and cytosolic factions, respectively).

TRIB3 had been previously implicated in the regulation of the PI3K/AKT axis, a signaling pathway that plays a relevant role in the control of BC generation and progression [36]. Therefore, we asked whether the above-described dual effect of the modulation of TRIB3 levels in luminal BC cells could be associated with changes on the activity of this key signaling route. In line with this idea, TRIB3 silencing decreased (Figure 2a) whereas TRIB3 overexpression enhanced (Supplementary Figure S3b) AKT phosphorylation on Ser473 of luminal A BC cell lines. Conversely knock-down of TRIB3 in luminal B BC cell lines induced the phosphorylation of AKT (Figure 2b), while enhanced expression of this pseudokinase diminished it (Supplementary Figure S3b). In line with our previous observations showing that TRIB3 can regulate the MTORC2/AKT/FOXO axis [29], these changes were selective of AKT Ser473 but not Thr308 (Figure 2c and Supplementary Figure S3c), and occurred in parallel with the phosphorylation of FOXO (Figure 2d). Altogether, these results indicated that TRIB3 plays an opposite role in the regulation of the PI3K/AKT pathway in luminal A and luminal B BC cell lines. We verified that these differences were not due to a distinct subcellular localization of TRIB3 in the two types of luminal BC cell lines (Figure 2e,f). Likewise, we confirmed by co-immunoprecipitation that AKT and TRIB3 interacted in both MCF7 and BT474 cells (Figure 2g). Since the other members of the tribbles family (tribbles pseudokinases 1 and 2, TRIB1 and TRIB2) have been shown to interact and potentially modulate the activity of TRIB3 [42], we analyzed their expression and subcellular localization in our cells. As shown in Figure 2h, we did not find any significant differences in those parameters between luminal A-like and luminal B-like cell lines that could justify their different behavior. Therefore, we postulated that the dual role played by TRIB3 in the regulation of luminal BC cell lines through the PI3K/AKT pathway might be determined by the cellular context and the presence of different kinase-signaling features between these two different BC molecular subtypes.

### 3.3. TRIB3 and HER2 Are Functionally Associated in Luminal B BC Cell Lines

**Figure 3:**
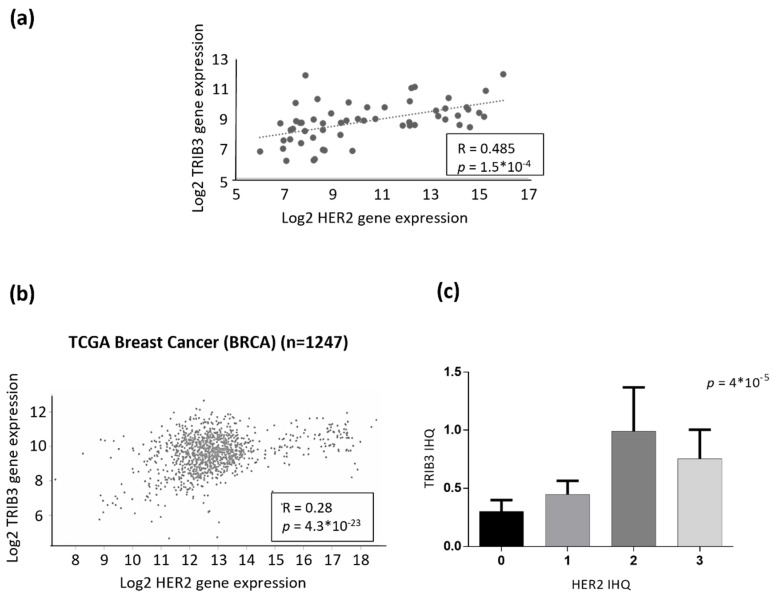
TRIB3 and HER2 expression exhibit a positive correlation in breast cancer. (**a**) Bioinformatic analysis of the correlation between TRIB3 and HER2 mRNA levels in a panel of breast cancer cell lines (RNA seq data were obtained from the GSE48216 dataset). (*n* = 56; *p* and R were calculated using Spearman’s correlation coefficient). (**b**) Bioinformatic analysis of the correlation between TRIB3 and HER2 mRNA levels in breast cancer patients (RNA seq data were obtained from the TGCA Breast cancer dataset using the UCSC Xena platform) (*n* = 1247; *p* and R were calculated using the Spearman correlation coefficient). (**c**) Correlation between TRIB3 protein levels (as determined by TRIB3 immunostaining and the subsequent assignment of the corresponding H value) with respect to HER2 levels (pathologically classified as 0-1-2-3 according to the level of expression of this receptor) in a tissue microarray that was generated with samples that were derived from luminal breast cancer patients (*n* = 159; *p* was calculated using the Spearman’s correlation coefficient).

**Figure 4:**
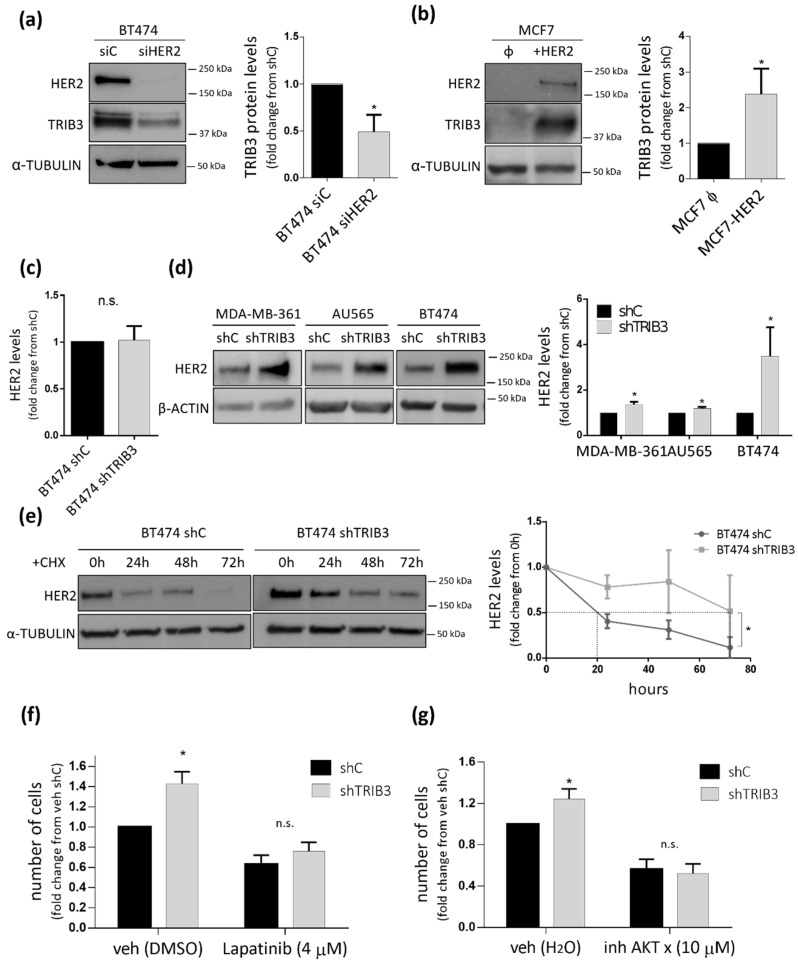
TRIB3 and HER2 mutually regulate their levels. (**a**) The effect of HER2 silencing (siHER2) or transfection with a control siRNA (siC) on TRIB3 levels of BT474 cells. Left panel: The Western blot images of a representative experiment are shown. Right panel: The data correspond to the optical density values in arbitrary units for each experimental condition normalized with respect to the loading control (α-TUBULIN) and are expressed as the mean fold change ± SEM with respect to siC (*n* = 5; * *p* < 0.05 by using the Wilcoxon test). (**b**) The effect of HER2 overexpression (+HER2) or transfection with an empty vector (Φ) on TRIB3 levels of MCF7 cells. Left panel: The Western blot images of a representative experiment are shown. Right panel: The data correspond to the optical density values in arbitrary units for each experimental condition normalized with respect to the loading control (α-TUBULIN) and are expressed as the mean fold change ± SEM with respect to the empty vector-transfected cells (Φ) (*n* = 5; * *p* < 0.05 by using the Wilcoxon test). (**c**) Effect of TRIB3 silencing on HER2 mRNA (as determined by qRT-PCR) levels of BT474 cells. The data correspond to HER2 mRNA normalized with respect to the loading control (GAPDH) and are expressed as the mean fold change ± SEM with respect the shC condition (*n* = 3). (**d**) The effect of TRIB3 stable knock-down on HER2 protein (as determined by Western blot) of MDA-MB-361, AU565, and BT474 cells. Left panel: The Western blot images of a representative experiment are shown. Right panel: The data correspond to the optical density values in arbitrary units for each experimental condition normalized with respect to the loading control (β-ACTIN) and are expressed as the mean fold change ± SEM with respect to the shC condition (*n* = 7; * *p* < 0.05 by using the Wilcoxon test). (**e**) The effect of TRIB3 silencing and incubation with the protein synthesis inhibitor cycloheximide (10 µM, CHX) on HER2 protein levels of BT474 cells at different timepoints. Left panel: The Western blot images of a representative experiment are shown. Right panel: The data correspond to the optical density values in arbitrary units for each experimental condition normalized with respect to the loading control (α-TUBULIN) and are expressed as the mean fold change ± SEM with respect to 0 h timepoint (*n* = 3; * *p* < 0.05 with respect to shC cells; using the two-way ANOVA test). (**f**) The effect of treatment with Lapatinib (4 μM, 48 h) or vehicle (veh, DMSO) on the number of BT474 shC and shTRIB3 cells (as estimated by crystal violet staining). The data correspond to the absorbance at 570 nm and are expressed as the mean fold change ± SEM with respect to veh-treated shC cells (*n* = 5; * *p* < 0.05 with respect to shC cells; using the two-way ANOVA test). (**g**) The effect of treatment with the AKT X inhibitor (10 μM, 48 h) or vehicle (veh, H_2_O) on the number of BT474 shC and shTRIB3 cells (as estimated by crystal violet staining). Data correspond to the absorbance at 570 nm and are expressed as the mean fold change ± SEM with respect to veh-treated shC cells (*n* = 6; * *p* < 0.05, with respect to the shC cells; using the two-way ANOVA test).

One of the fundamental molecular differences between luminal A and luminal B BC cells is that the latter expresses the HER2 oncogene. Therefore, we wondered whether the role of TRIB3 in luminal BC cell lines could be determined by the presence of alterations in this tyrosine kinase receptor. Analysis of HER2 and TRIB3 expression in our panel of luminal BC cell lines showed a clear positive correlation between the expression of these two proteins (Supplementary Figure S3a). Moreover, bioinformatic analyses revealed the existence of a strong correlation between TRIB3 and HER2 mRNA levels in a wide panel of human breast cancer cell lines (Figure 3a) as well as in the TGCA BC database (Figure 3b). Furthermore, analysis of TRIB3 protein expression in a tissue microarray of luminal BC patients containing HER2+ and HER2− tumors (see materials and methods section and below for a more precise description of these samples) confirmed the correlation between the intensity of TRIB3 staining and HER2 expression at the protein level (Figure 3c). To investigate the existence of a mechanistic connection between these two events we selected BT474 cells as a representative model of luminal B cells that were expressing high levels of HER2 and MCF7 as a model of luminal A cells. As shown in Figure 4a, knock-down of HER2 in BT474 cells decreased TRIB3 levels whereas HER2 overexpression in MCF7 cells enhanced the expression of this pseudokinase (Figure 4b). These observations supported the idea that HER2 positively regulates TRIB3 expression in BC cell lines.

Next, we analyzed whether TRIB3 plays a role on the regulation of HER2 levels in luminal B (HER2+) BC cell lines. Of note, we found that TRIB3 silencing induced a significant upregulation of HER2 at the protein but not the mRNA level (Figure 4c,d). Therefore, we hypothesized that TRIB3 could regulate the stability of HER2 at a posttranslational level. To prove this idea, we performed experiments in which TRIB3 was ablated in the presence of the protein synthesis inhibitor cycloheximide and found that HER2 protein levels were increased (Figure 4e). This result indicated that TRIB3 decreases HER2 levels by reducing its stability and led us to postulate that the increased proliferation and enhanced AKT activation that was previously observed in TRIB3-silenced luminal B BC cell lines could be a result of HER2 signaling hyperactivation. In agreement with this possibility, the pharmacological blockade of HER2 (Figure 4f) or AKT (Figure 4g) prevented the TRIB3 genetic inhibition-induced increase of BT474 proliferation. Altogether these results point to the existence of feedback regulatory mechanisms in luminal B BC cells where HER2 positively regulates TRIB3 expression whereas this pseudokinase negatively regulates the stability of HER2.

### 3.4. Analysis of TRIB3 Protein Levels in a Wide Cohort of Breast Cancer Patients Unravels Its Potential Role as a Biomarker of Good Prognosis in Luminal BC

**Figure 5:**
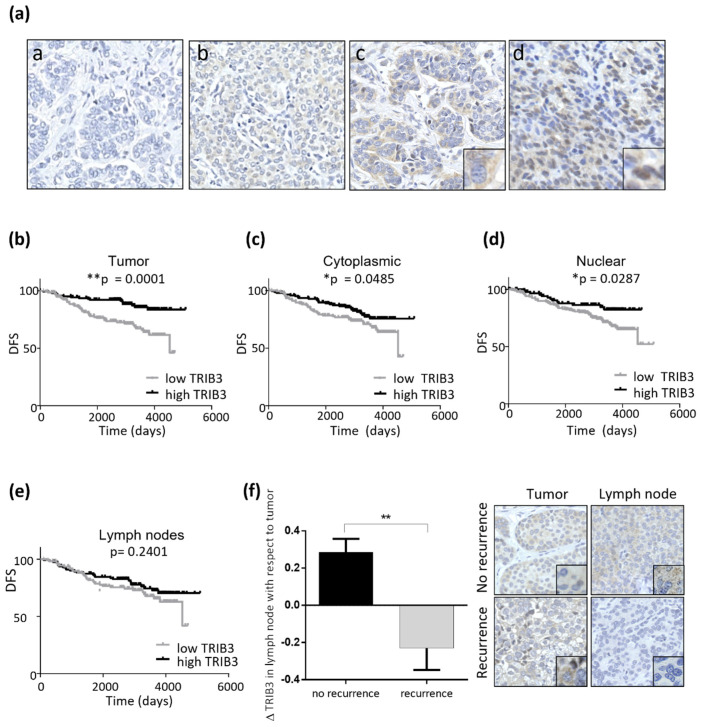
High TRIB3 protein levels are associated to good prognosis in luminal breast cancer patients. (**a**) The representative images (40 ×) of the pattern of TRIB3 immunostaining in samples from a tissue microarray of luminal breast cancer patients (**a**: negative/non-detectable, **b**: low intensity, **c**: medium intensity cytoplasmatic staining (see inset–6X digital amplification—for details), and **d**: high intensity showing a mainly nuclear staining pattern (see inset–6X digital amplification—for details). (**b**–**d**) Kaplan-Meier representation of disease-free survival (DFS) according to the intensity of TRIB3 immunostaining in the primary breast tumor. Panel b considers TRIB3 protein levels irrespective of their cytoplasmic or nuclear localization whereas panels c and d correspond to the intensity of the cytoplasmic (**c**) or nuclear (**d**) TRIB3. (**e**) Kaplan-Meier curves representation of DFS according to the intensity of TRIB3 immunostaining in the affected lymph nodes. The samples were stratified as TRIB3 high- or low-levels by using the median value (*n* = 291; * *p* < 0.05, ** *p* < 0.01 by log-rank test Mantel-Cox). (**f**) Left panel: The correlation between TRIB3 levels in the affected lymphatic node with respect to the primary tumor and the recurrence of the disease (*n* = 291; ** *p* < 0.01 by Kolmogorov-Smirnov Test). Right Panel: microphotographs (40 ×) of TRIB3 staining of the primary tumor and affected lymphatic node see inset–6 × digital amplification—for details) of two patients that were representative of the non-recurrence and recurrence groups, respectively.

Considering the previous observations, we wondered whether TRIB3 expression had a differential predictive value in luminal A and B breast cancer. As discussed above, previous studies had shown that TRIB3 mRNA and protein levels do not always correlate in human breast cancer patient samples [31,32]. Therefore, we opted to analyze TRIB3 protein levels in a Tissue microarray that was generated from a prospective cohort of 291 patients classified as luminal (hormone-dependent) breast tumors (Table S1). Of note, these patients had at least one affected lymph node at the time of diagnosis and, therefore, were potentially prone to progress to an advanced disease. The tissue microarray contained samples from tumor tissue and, when available, the affected lymph nodes. These samples had been taken during the first surgery and prior to the commencement of the treatments. The patients were treated with hormonal therapy (289), conventional chemotherapy (225), and radiotherapy (222). In addition, a fraction of patients progressed to recurrent (71) or metastatic (64) disease (Table S1). An initial analysis of TRIB3 expression revealed the existence of differences in the intensity and the subcellular distribution of the staining among the different samples. Thus, TRIB3 expression could be detected in both the nucleus and the cytoplasm of tumor cells (Figure 5a). H scores were assigned based on the percentage of TRIB3-positive cells and the intensity of the staining for each of the samples that were subsequently stratified as “high TRIB3” and “low TRIB3” (see materials and methods for additional information).

A detailed analysis of the clinical features of each of the patients revealed that “high TRIB3” expression was associated with a more prolonged disease-free survival, an effect that was maintained when nuclear or cytoplasmic localization of the protein in the primary tumor was considered (Figure 5b–d). We did not find a statistically significant correlation between TRIB3 expression in the lymph node and DFS (Figure 5e). However, when we considered the expression of TRIB3 in the lymph node and in the primary tumor we found that a higher ratio (implying an increased or maintained expression of TRIB3 in the lymph node) was associated with a lower recurrency of the disease (Figure 5f). These observations led us to conclude that a higher expression of TRIB3 could be a factor of good prognosis in luminal BC.

### 3.5. TRIB3 Expression Is Associated to a Better Response to Therapy in Luminal BC

**Figure 6:**
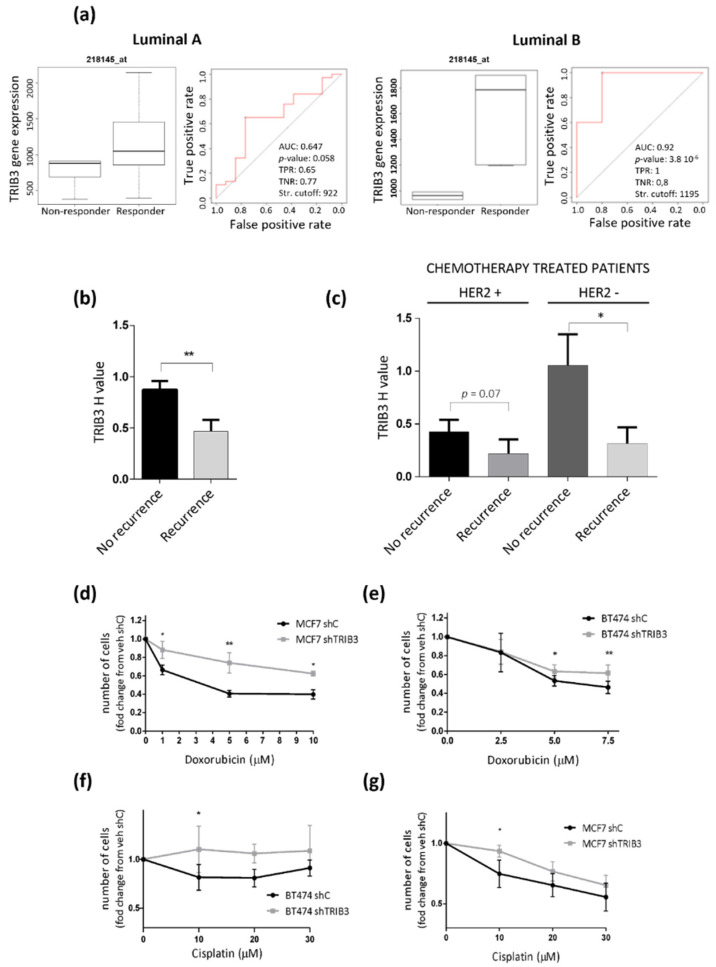
High TRIB3 levels correlate with better response to therapy in both HER2- and HER2+ luminal breast cancer. (**a**) The bioinformatic analysis of the association between TRIB3 mRNA levels and complete pathological response to endocrine therapies in ER-positive luminal A (left panel) and luminal B (right panel) breast cancer patients. The receiver operating characteristic (ROC) curves are shown. The area under the curve (AUC), *p* value, false positive rate (FPR), and true positive rate (TPR). (**b**,**c**) The correlation between TRIB3 protein levels (as determined by TRIB3 immunostaining) and recurrence after treatment with chemotherapy in a TMA of luminal breast cancer patients. Note that all patients had been initially treated with endocrine therapy. The data correspond to the H-value quantification of TRIB3 levels in the primary tumors and are expressed as the mean H value ± SEM for each set of patients. Panels b and c show information relative to all of the patients that were subjected to chemotherapy irrespective of HER2 status (panel B, *n* = 150) or separated according to HER2 expression (Panel c, *n* = 32 for HER2+ and *n* = 118 for HER2-). ** *p* < 0.01 and * *p* < 0.05 with respect to non-recurrent patients by using a T-test with Welch correction. (**d**–**g**) The effect of TRIB3 silencing (shTRIB3) and treatment with Doxorubicin (**d**,**e**) or Cisplatin (**f**,**g**) on the number of BT474 (**e**,**f**) and MCF7 (**d**,**g**) cells (as estimated by crystal violet staining). The data correspond to the absorbance at 570 nm and are expressed as the mean fold change ± SEM with respect to veh-treated shC cells (*n* = 3–5; ** *p* < 0.01 and * *p* < 0.05 with respect to shC cells; using the two-way ANOVA test).

The above finding that TRIB3 is associated with a higher DFS of luminal BC patients was apparently at odds with the previously discussed observation that TRIB3 overexpression enhances luminal A-like BC cell lines proliferation. We, therefore, asked whether TRIB3 might play a role in the regulation of the sensitivity to anticancer treatments as this is a factor that has a decisive impact in the progression of the disease. Since most luminal BC patients are initially treated with endocrine therapy, we first analyzed the correlation between the response to these therapies (tamoxifen or aromatase inhibitors) and TRIB3 expression in databases from published studies [43]. Interestingly, we found that there is a strong association between high TRIB3 expression and a pathological complete response to endocrine therapies in luminal A and luminal B BC patients (Figure 6a). Encouraged by this observation we next analyzed in our TMA whether TRIB3 expression was associated with the efficacy of the anticancer treatments. Most of the patients from this cohort were treated with endocrine therapy followed by treatment with chemotherapy and we found that higher expression of TRIB3 correlated with a higher efficacy of chemotherapy (defined as the lack of progression to an advanced or metastatic disease) within all of the cohort, and specifically within HER2- patients (Figure 6b,c). To confirm these observations in a cellular model we evaluated the effect of two standard anticancer treatments (doxorubicin and cisplatin) that were used in the treatment of advanced luminal BC on the viability of MCF7 and BT474 cells in which TRIB3 had been silenced. As shown in Figure 6d–g, knock-down of TRIB3 rendered both MCF7 and BT474 cells more resistant to the treatment with these agents further supporting the idea that enhanced TRIB3 expression may facilitate the response to these therapies in both HER2- and HER2+ luminal BC cells.

## 4. Discussion

Previous work by different groups has shown that TRIB3 can exert both oncogenic [18,19,20,21,22,23,24] and onco-suppressive [16,25,26,27,28,29,30] functions in different cellular and animal models of cancer. In this study, we showed that TRIB3 plays a dual role in the regulation of luminal breast cancer cell lines. Thus, our observations support the notion that enhanced expression of TRIB3 promotes the proliferation of luminal A while driving the opposite effect in luminal B (HER2+) breast cancer cell lines. Furthermore, our results indicate that this effect of TRIB3 genetic manipulation is mediated by changes in the activity of the AKT/FOXO axis, suggesting that this pseudokinase regulates the AKT pathway differently in luminal A and luminal B breast cancer cell lines. Noteworthy previous studies had shown that TRIB3 promotes tumor development and resistance to therapy in triple negative breast cancer models by regulating the NOTCH pathway [33] or the stability of SOX2 via AKT inhibition and stabilization of FOXO1 [34]. In contrast, we had previously shown that TRIB3-mediated inhibition of AKT and the subsequent activation of FOXO transcription factors reduces cell proliferation and survival and inhibits tumor growth in different tumor models, including breast cancer cell lines [29]. In this study we found that TRIB3 and AKT interact with each other at a similar extent in both types of luminal breast cancer cell lines suggesting that the mechanisms that determine this dual role of TRIB3 in the regulation of AKT and AKT downstream targets may rely on additional factors. Thus, the molecular alterations that are characteristic of each breast cancer subtype may directly impact on the capacity of TRIB3 to regulate this and other oncogenic signaling pathways. Specifically, our findings point to the existence of a crossed regulation between HER2 and TRIB3 in luminal B breast cancer cells. Thus, HER2 oncogenic signaling enhances TRIB3 expression whereas TRIB3 negatively regulates the stability of HER2. These observations indicate that, in this context, there is a feedback regulatory mechanism by which TRIB3 contributes to modulate HER2 oncogenic signaling. Thus, alterations involving a decreased expression of TRIB3 in this BC subtype would enhance the proliferative and tumorigenic [29] capacity of these cells. The precise molecular mechanisms by which TRIB3 regulates the stability of HER2 need to be investigated in further detail. In any case, it is worth pointing that it has been previously described that TRIB3 controls the activity of several ubiquitin E3 ligases via its COP1 domain [44] and that the levels of HER2 are controlled via its ubiquitylation [45,46,47]. Therefore, it is conceivable that a similar mechanism could operate in this context. Nevertheless, other possible mechanisms of TRIB3 regulation such as changes in the miRNA pattern [48,49] or the interaction with other members of the tribbles pseudokinases family [42] may also impact the regulation of the levels or the activity of TRIB3 and should be considered.

Although the above discussed experiments that were performed in cellular modes of breast cancer indicate that the modulation of TRIB3 expression produces an opposite effect on luminal A and luminal B (HER2+) breast cancer cell lines, the findings that are presented in this report strongly support the idea that high TRIB3 protein levels could be a biomarker of good prognosis in all luminal breast cancer patients (the most frequent breast cancer subtype) irrespective of whether they are luminal A or luminal B. Previous studies on the potential use of TRIB3 as a cancer biomarker were scarce and indicated that TRIB3 mRNA and protein levels do not show a good correlation in breast cancer patients [31,32]. In this work, the analysis of TRIB3 expression in a tissue microarray that was generated with samples from patients of luminal breast cancer revealed a clear correlation between high TRIB3 expression and higher DFS in this cohort that contained a majority of HER2− tumors. Moreover, the maintenance of TRIB3 expression in the affected lymph nodes with respect to the primary tumor at the time of diagnosis is associated with a lower frequency of recurrence in these patients. Although the cohort of patients included in this study is relatively small and should be ideally expanded with the analysis of additional samples from other datasets of luminal breast cancer patients, these results suggest that evaluation of TRIB3 protein expression in samples from luminal breast cancer patients could have prognostic interest and should be further investigated.

One of the key factors that determines the evolution of cancer patients is the sensitivity to different therapies. All luminal breast cancer patients are initially treated with endocrine therapies that, depending on the stage of the tumor at the time of diagnosis, are complemented with radiotherapy, chemotherapy, or targeted therapies. Interestingly in our study we found that TRIB3 expression is associated with a better response to endocrine therapies in both luminal A and luminal B breast cancer patients. Moreover, high TRIB3 expression was associated with a lower frequency of recurrences in patients from the tissue microarray study that had been subjected to endocrine therapy + chemotherapeutic treatments irrespective of whether they were luminal A (HER2−) or luminal B (HER2+). These observations support the idea that the increased expression of TRIB3 sensitizes the tumors to the treatment with the therapies that are usually employed in the management of luminal breast cancer, which could help to explain why TRIB3 high expression is associated with good prognosis in both luminal A and luminal B breast cancer patients. Interestingly, different studies have shown that TRIB3 upregulation plays a crucial role in the mechanism of action of several anticancer agents [50,51,52,53]. From these results, it is tempting to speculate that the strategies that are aimed at driving TRIB3 upregulation could act as sensitizers to other therapies and therefore be beneficial in the treatment of luminal breast cancer patients when used in combination with current anticancer treatments. Exploring the efficacy of some of these combinations in preclinical models of luminal breast cancer would be of great interest as it might potentially help to set the basis for the development of more effective anticancer treatments for the management of advanced luminal breast cancer.

## 5. Conclusions

Findings presented in this work support the idea that TRIB3 enhanced expression is associated with good prognosis and a better response to therapy in luminal breast cancer patients. Our data also indicate that TRIB3 inhibits the HER2 pathway in Luminal B (HER2+) breast cancer cells. These observations warrant further exploration of TRIB3 as a potential biomarker of good prognosis in luminal breast cancer. 

## Data Availability

The data presented in this study are available in this article (and supplementary material) and on request from the corresponding author.

## Supplementary Materials

The following are available online at https://www.mdpi.com/article/10.3390/cancers13215307/s1, Figure S1: Correlation between TRIB3 mRNA levels and DFS in breast cancer patients, Figure S2: Differential role of TRIB3 in the regulation of luminal A and luminal B breast cancer cell lines, Figure S3: TRIB3 regulates differently the AKT pathway in luminal A and luminal B breast cancer cell lines, Table S1: Description of clinical features of the patients included in the TMA, File S1: Original Western blot images.
