# Supplementary material for: The Pseudokinase TRIB3 Negatively Regulates the HER2 Receptor Pathway and Is a Biomarker of Good Prognosis in Luminal Breast Cancer
Source: Cancers (Basel). 2021 Oct 22;13(21):5307. doi: 10.3390/cancers13215307 (PMC8582533; doi:10.3390/cancers13215307)

SUPPLEMENTARY INFORMATION

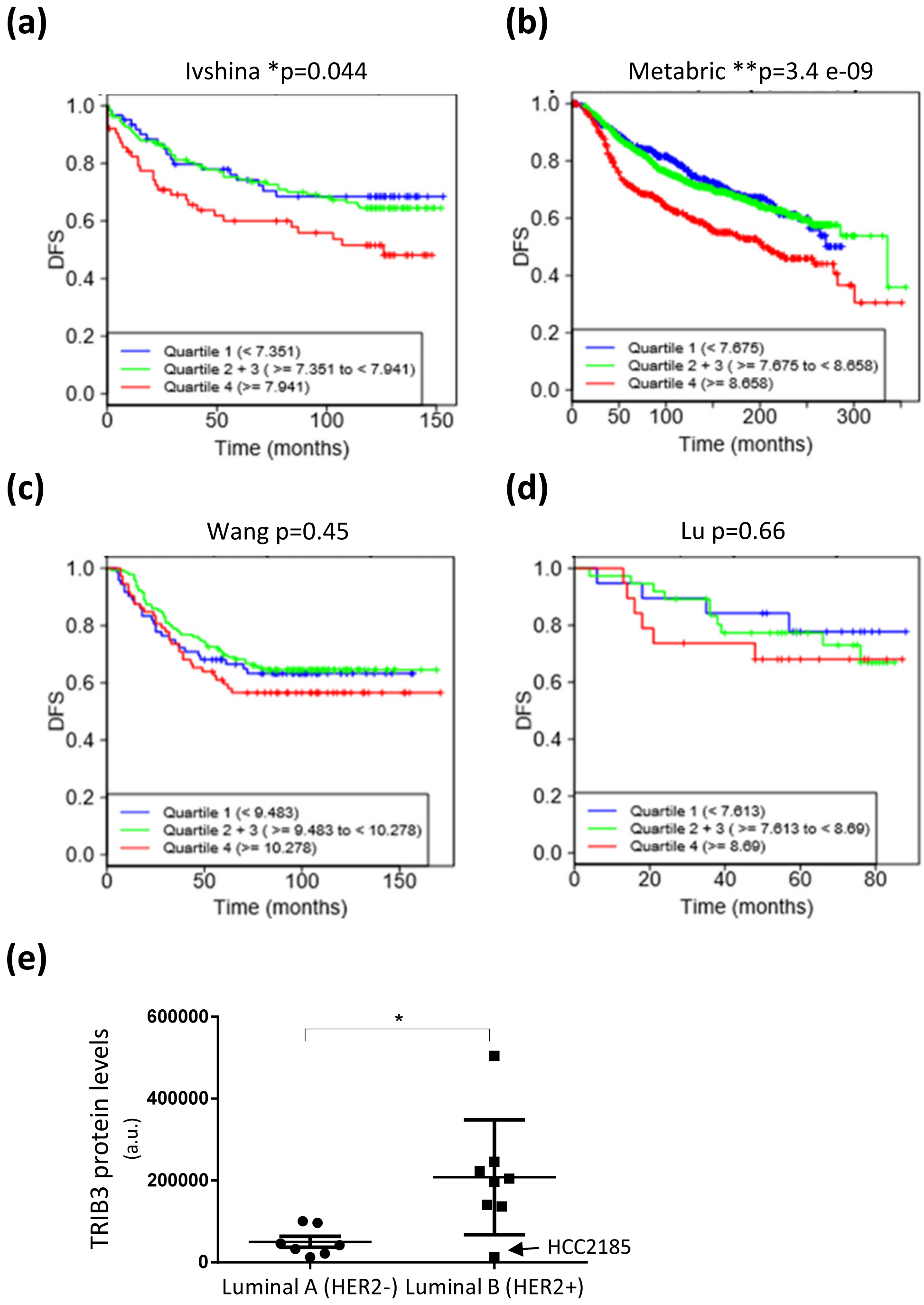

Supplementary Figure S1. Correlation between TRIB3 mRNA levels and DFS in breast cancer patients: (a-d) Kaplan-Meier representation of disease-free survival (DFS) in breast cancer patients according to TRIB3 mRNA levels stratified in quartiles (Cancertool platform was used to obtain data from different studies; (a) Ivshina (n= 249), (b) Metabric (n=980), (c) Wang (n = 286), (d) Lu (n=131); n=number of patient samples; Quartile 4 expression was different from quartile 1 (* p < 0.05 and ** p < 0.01) in the Ivshina and Metabric datasets. Log-rank text (Mantel-Cox). (e) Densitometric analysis of TRIB3 levels in luminal A (HER2-) and luminal B (HER2+) cells. Data correspond to the optical density values in arbitrary units for each experimental condition normalized with respect to HSP90 levels and are expressed as the mean ± SEM (n = 7-8; * p < 0.05; T-test with Welch correction). Note that HCC2185 cells exbibit low levels of HER2 and TRIB3

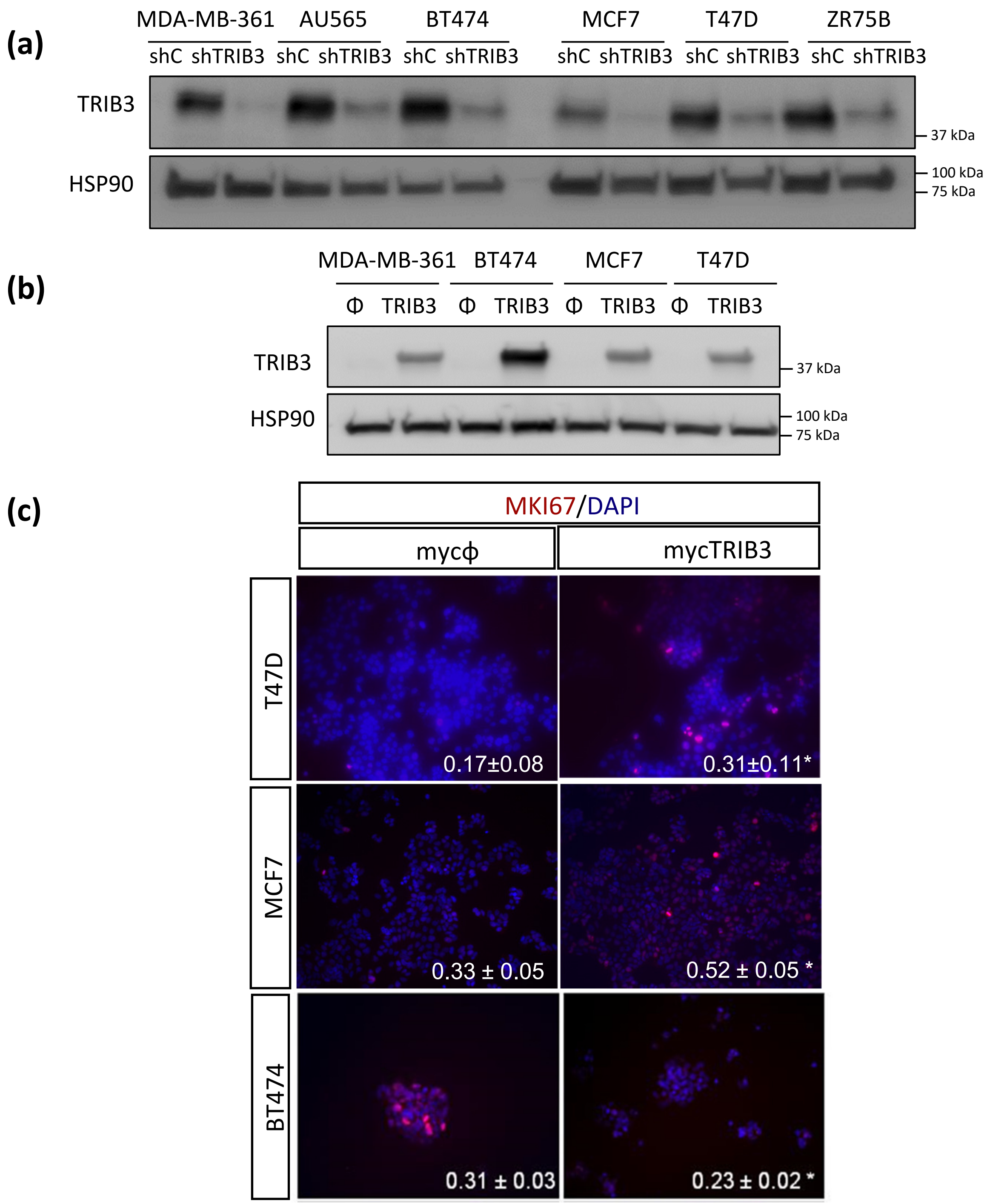
(a)
MDA-MB-361
AU565
BT474
MCF7
T47D
ZR75B
shC shTRIB3 shC shTRIB3 shC shTRIB3 shC shTRIB3 shC shTRIB3 shC shTRIB3
TRIB3
HSP90
37 kDa
100 kDa
75 kDa
(b)
MDA-MB-361
BT474
MCF7
T47D
Φ TRIB3 Φ TRIB3 Φ TRIB3 Φ TRIB3
TRIB3
HSP90
37 kDa
100 kDa
75 kDa
(c)
MKI67/DAPI
mycϕ
mycTRIB3
T47D
0.17±0.08
0.31±0.11*
MCF7
0.33 ± 0.05
0.52 ± 0.05 *
BT474
0.31 ± 0.03
0.23 ± 0.02 *

Supplementary Figure S2. **Differential role of TRIB3 in the regulation of luminal A and luminal B breast cancer cell lines.** (a) Effect of stable transfection with a TRIB3-selective shRNA smatpool (shTRIB3) or a control shRNA (shC) on TRIB3 proteins levels of different breast cancer cell lines. A representative Western blot experiment is shown (n = 10). (b) Effect of transient transfection with an empty vector (Φ) or a vector encoding myc-TRIB3 on TRIB3 protein levels of different breast cancer cell lines. A representative Western blot experiment is shown (n = 10). (c) Effect of TRIB3 expression on the proliferation (as determined by immunostaining with MKI67, red) of luminal breast cancer cell lines. Representative images of the immunofluorescence staining are shown. Nuclei were stained with DAPI (blue). Values in the lower right corner of each microphotograph correspond to the mean fraction ± SEM of the number of MKI67 positive cells with respect to the total number of cells. Ten fields per experimental condition were counted to carry out the quantifications (n = 4-5; * $p < 0.05$ with respect to empty vector-transfected cells; using the T test).

Orea-Soufi et al. Figure S3

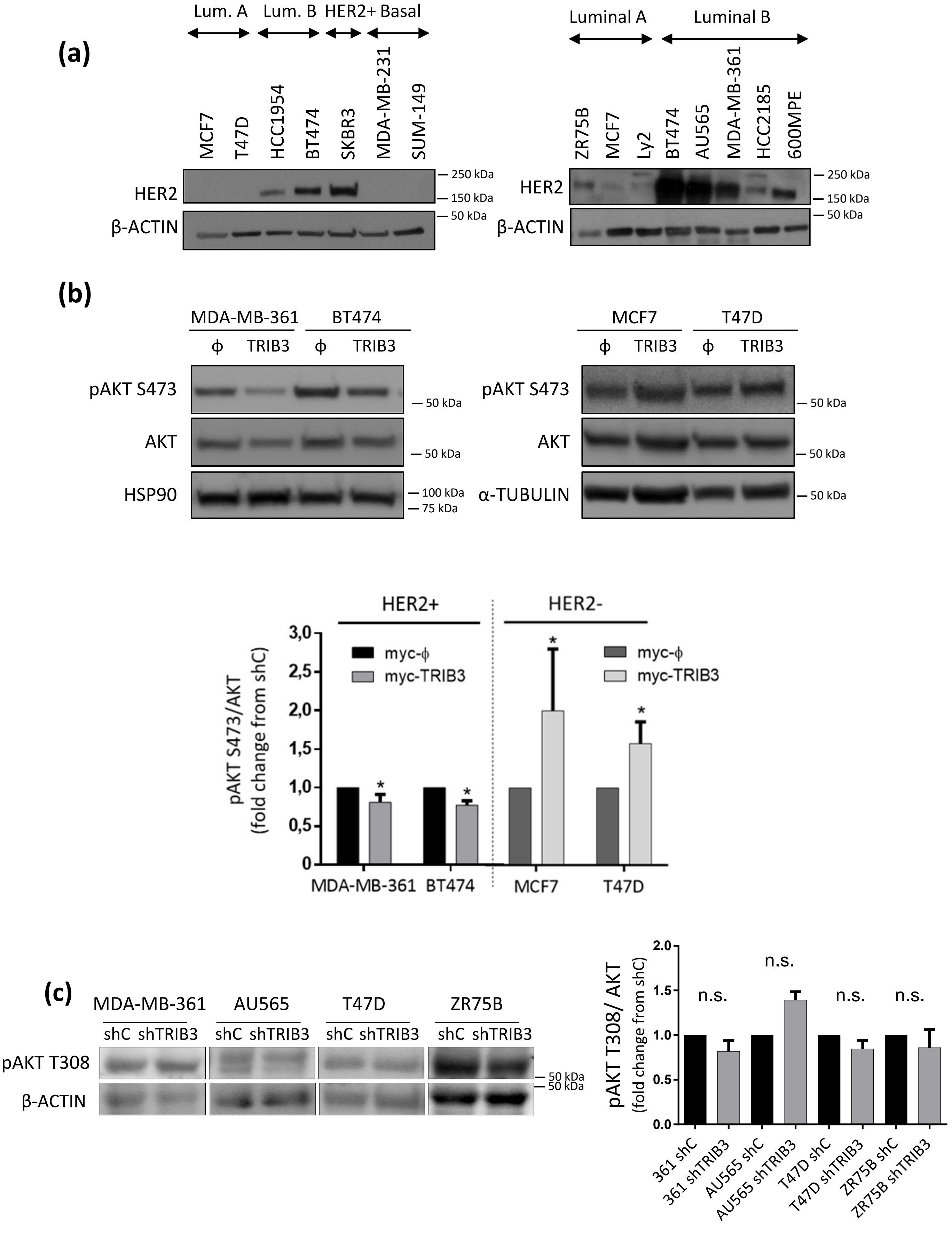

Supplementary Figure S3. TRIB3 regulates differently the AKT pathway in luminal A and luminal B breast cancer cell lines. (a) HER2 protein levels (as determined by Western blot) in a panel of cell lines representative of the different breast cancer subtypes (left panel) or of luminal (right panel) breast cancer. (b) Effect of TRIB3 overexpression on AKT phosphorylation at serine 473 of luminal B (upper left panel) and luminal A (upper right panel) breast cancer cell lines. Upper panels: Western blot images of a representative experiment are shown. Lower panel: Densitometric analysis of AKT phosphorylation at serine 473 (pAKT S473). Data correspond to the optical density values in arbitrary units for each experimental condition normalized with respect to the total AKT and HSP90 levels and are expressed as the mean fold change ± SEM with respect to empty vector-transfected cells (myc-ϕ) for each case (n = 6-12; * $p < 0.05$, with respect to myc-ϕ; Wilcoxon test). (c) Effect of TRIB3 silencing on AKT phosphorylation at threonine 308 of breast cancer cell lines. Left panel: Western blot images of a representative experiment are shown. Right panel: Densitometric analysis of AKT phosphorylation at threonine 308 (pAKT T308). Data correspond to the optical density values in arbitrary units for each experimental condition normalized with respect to B-ACTIN levels and are expressed as the mean fold change ± SEM with respect to shC for each case (n = 8; Wilcoxon test).

Supplementary Table S1. Description of clinical features of the patients included in the TMA.

Clinical Features		Number of patients
**Sex**	Male	3
	Female	198
**Histological grade**	1 (well differentiated)	39
	2 (moderately differentiated)	63
	3 (poorly differentiated)	78
**Recurrence**	No	217
	Yes	71
**Metastasis**	No	221
	Yes	64
**Treatments**	None	1
	Hormonotherapy	24
	Hormonotherapy + radiotherapy	40
	Hormonotherapy + chemotherapy	44
	Hormonotherapy + radiotherapy + cheemotherapy	181
**Molecular markers**		**Number of patients**
**ER**	Negative	0
	Positive	291
**PR**	Negative	47
	Positive	244
**HER2**	0	65
	1	66
	2	11
	3	17

ORIGINAL WB (File S1)

Fig. 1C (left panel) and S3A (HER2)

HER2

B-ACTIN

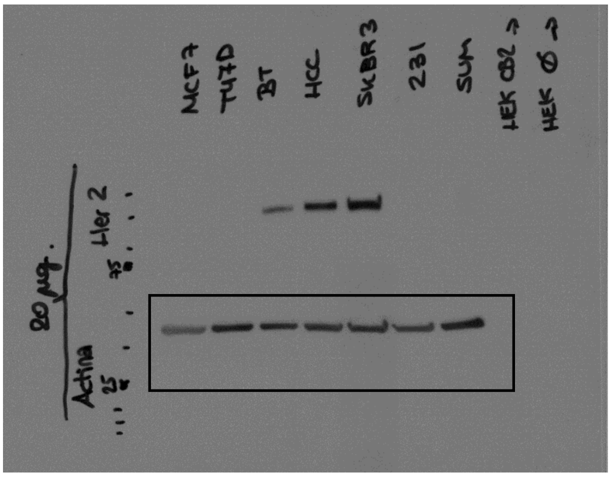

TRIB3

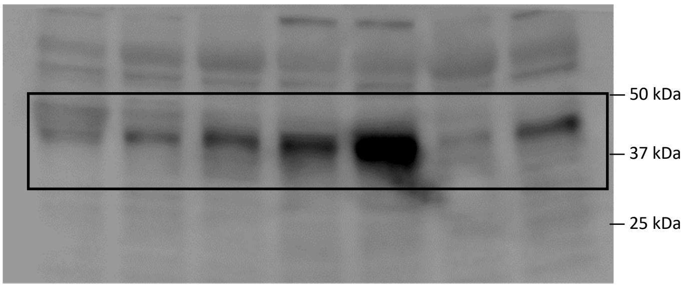

Fig. 1C (right panel) and S3A (HER2)

TRIB3

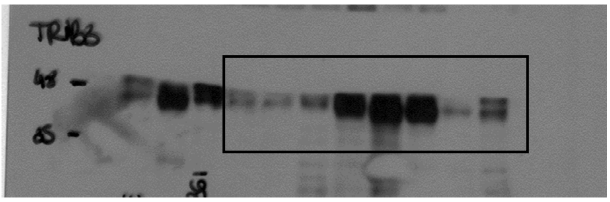

B-ACTIN

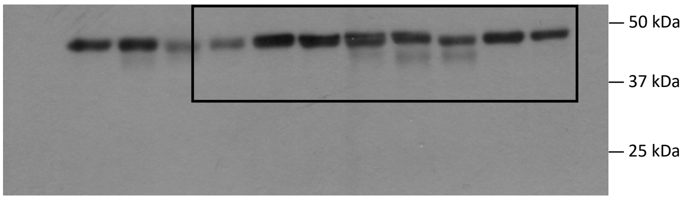

Fig. 2A

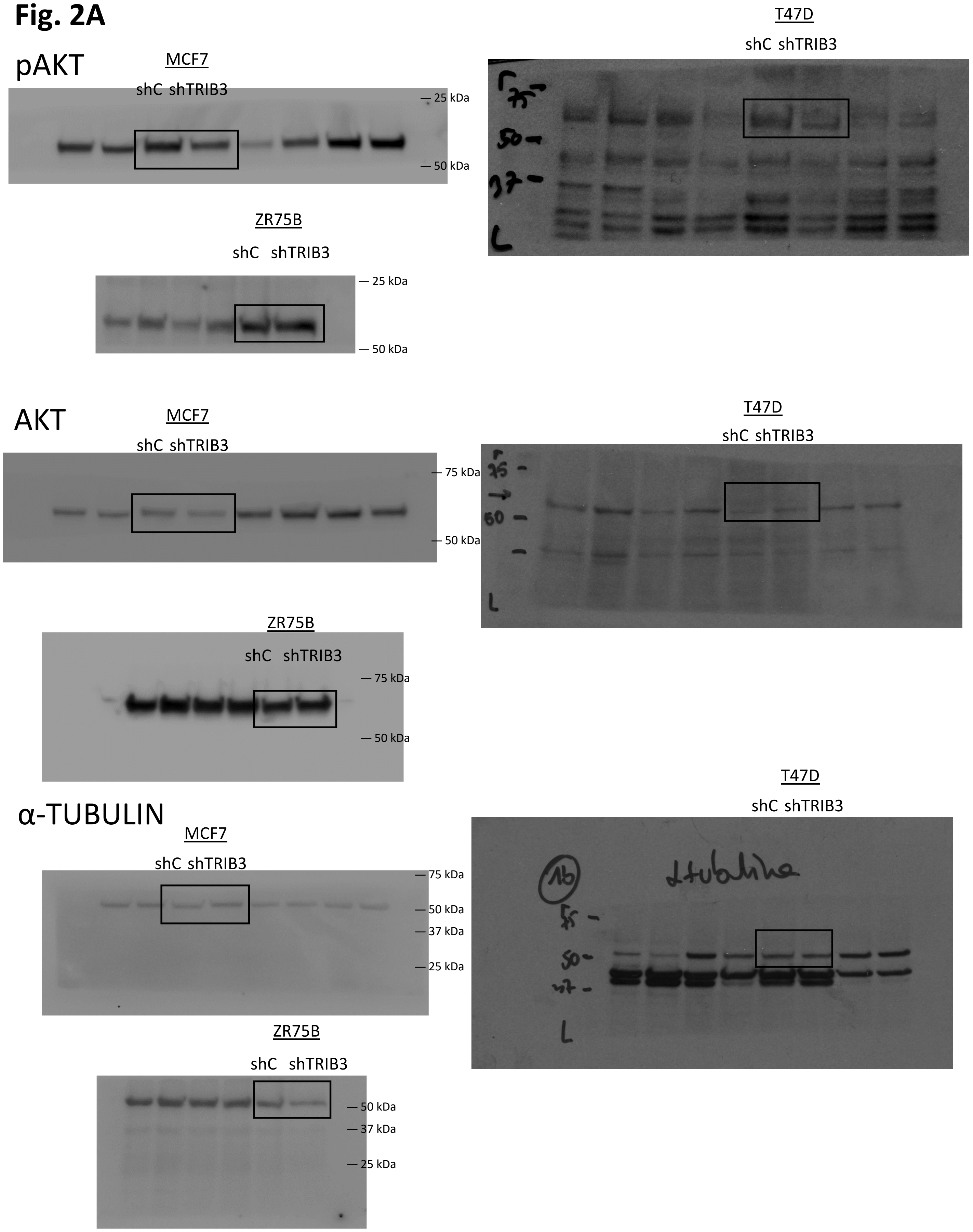
pAKT
MCF7
shC shTRIB3
25 kDa
50 kDa
T47D
shC shTRIB3
ZR75B
shC shTRIB3
25 kDa
50 kDa
AKT
MCF7
shC shTRIB3
75 kDa
50 kDa
T47D
shC shTRIB3
ZR75B
shC shTRIB3
75 kDa
50 kDa
α-TUBULIN
MCF7
shC shTRIB3
75 kDa
50 kDa
37 kDa
25 kDa
T47D
shC shTRIB3
ZR75B
shC shTRIB3
50 kDa
37 kDa
25 kDa

Fig. 2B

pAKT

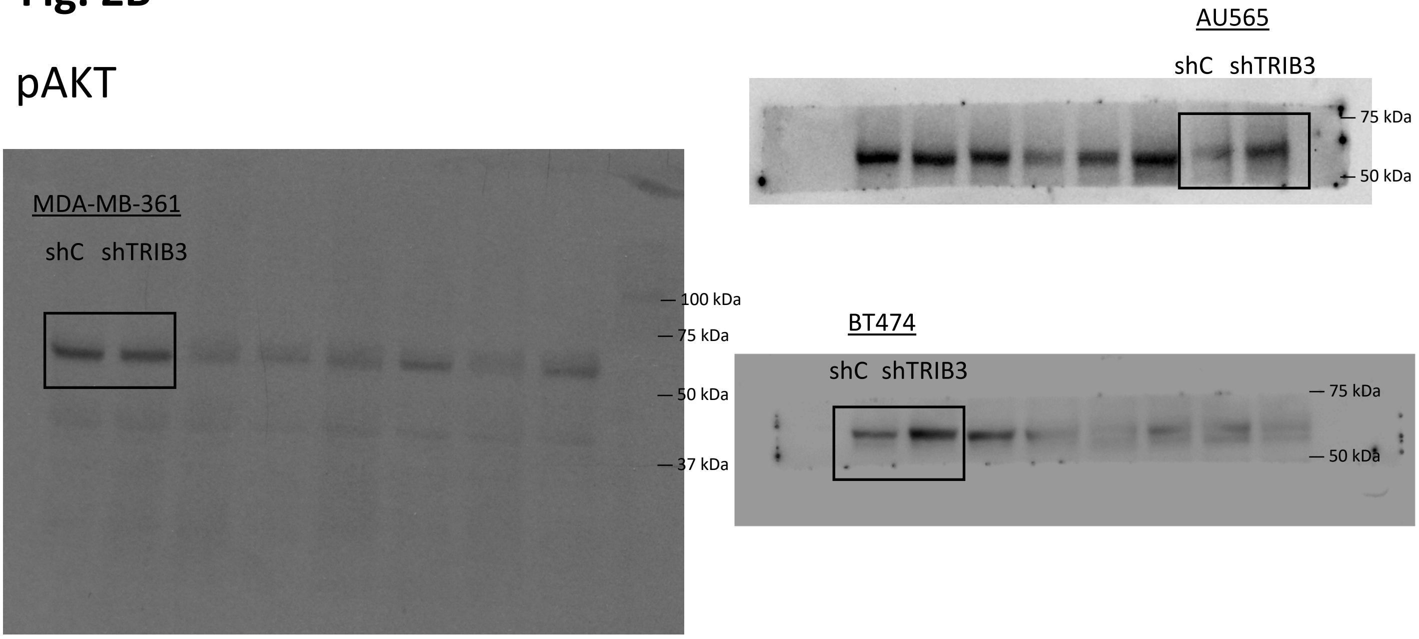

AKT

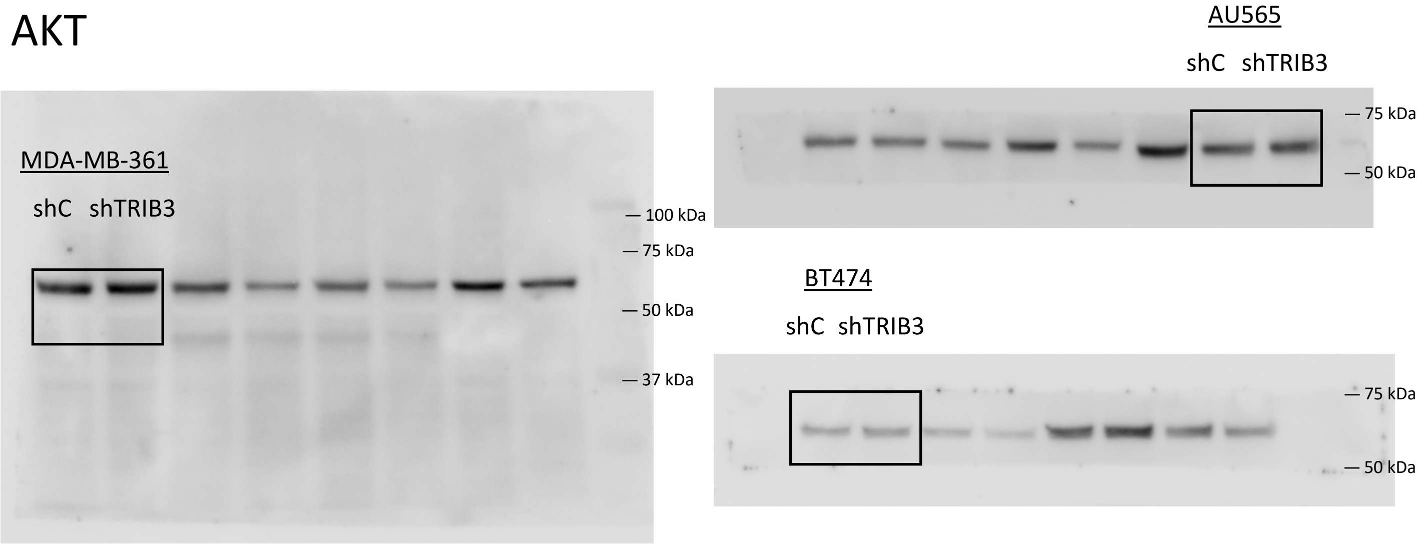

α-TUBULIN

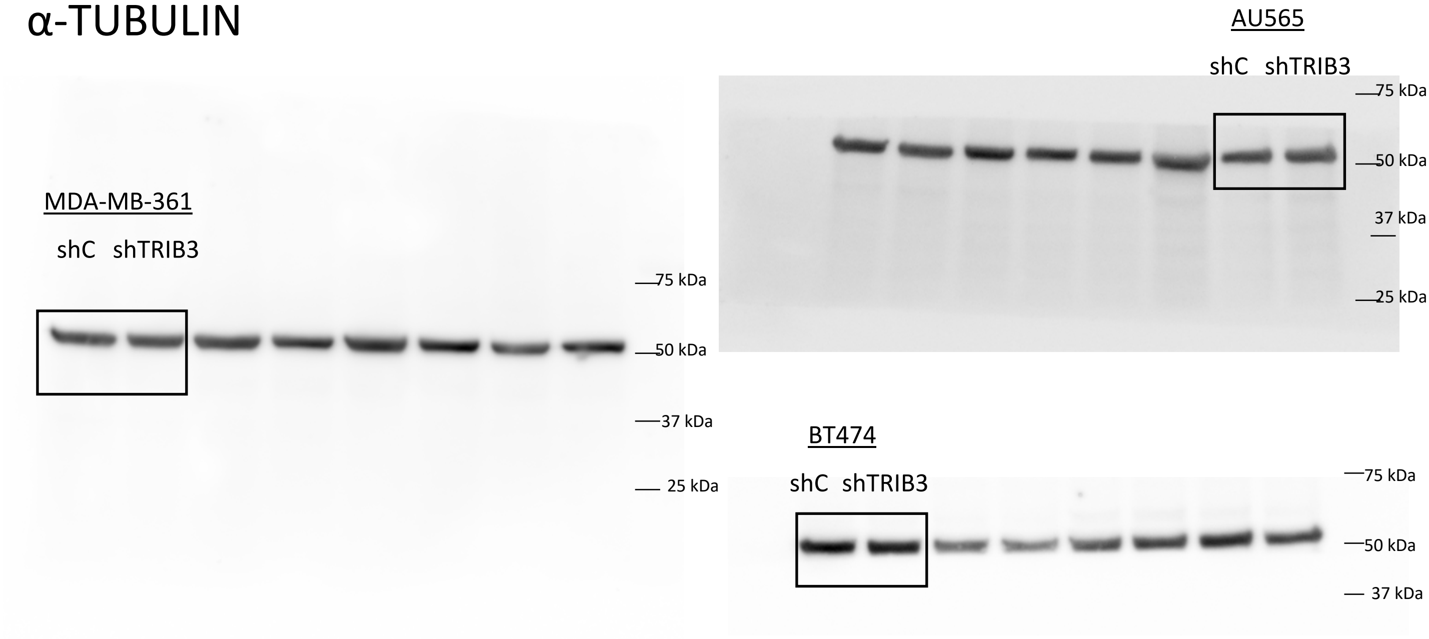

Fig. 2C

pAKT T308

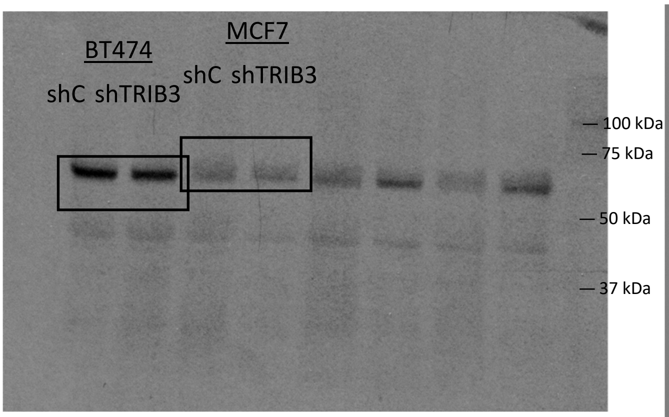

AKT

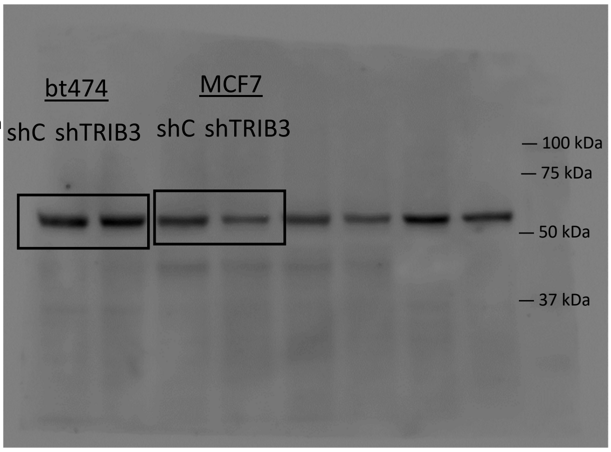

α-TUBULIN

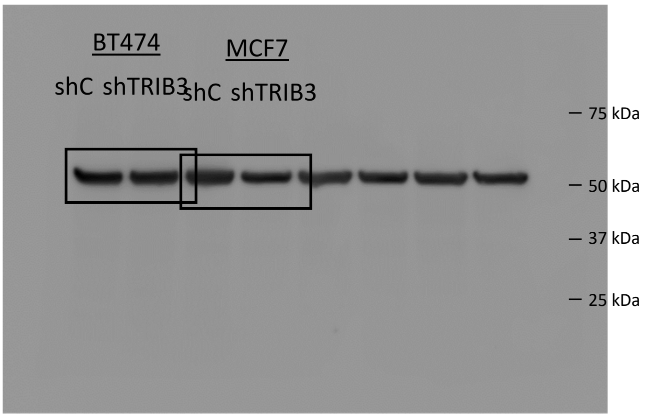

Fig. 2D

pFOXO

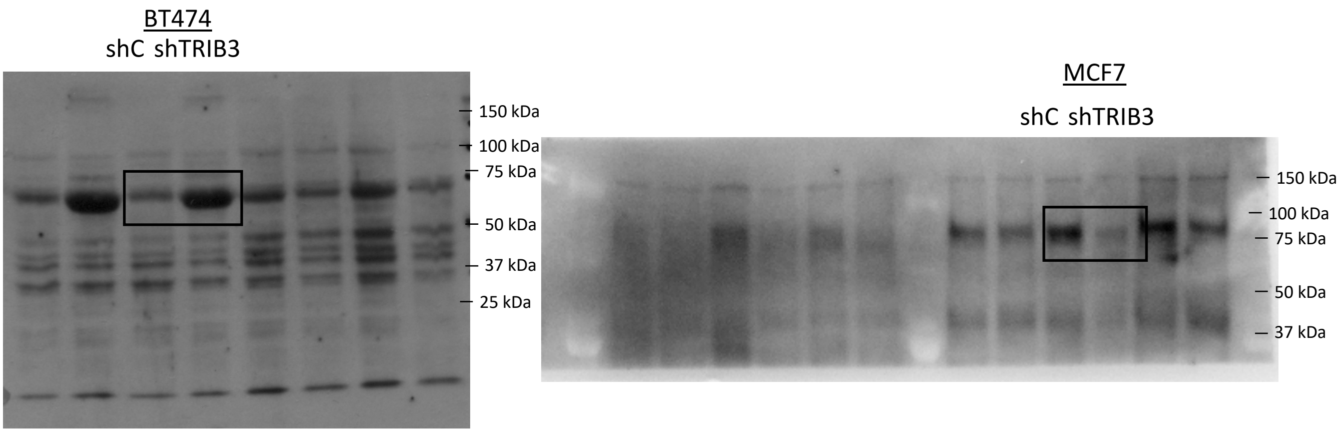

FOXO

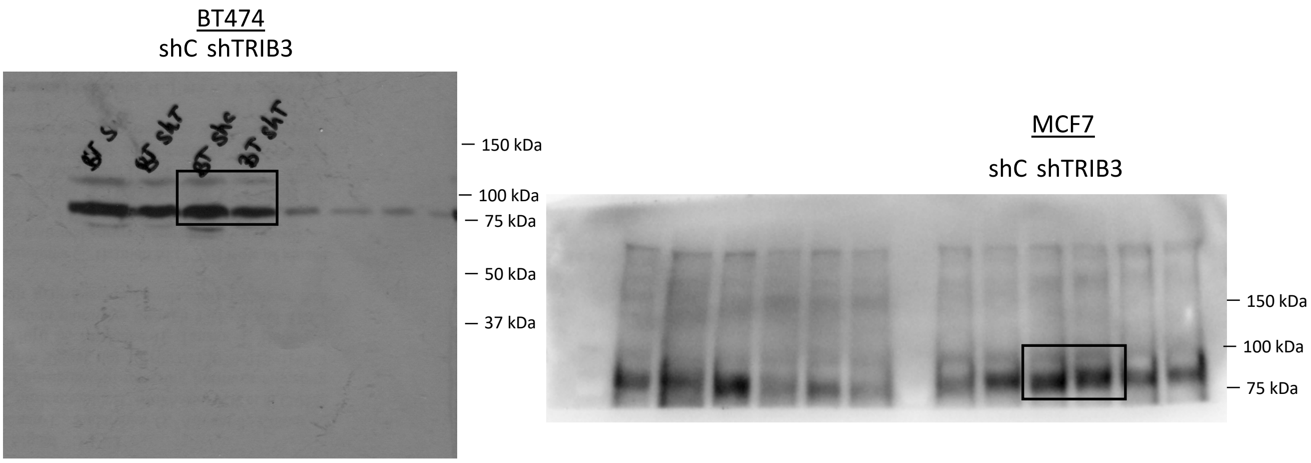

α-TUBULIN

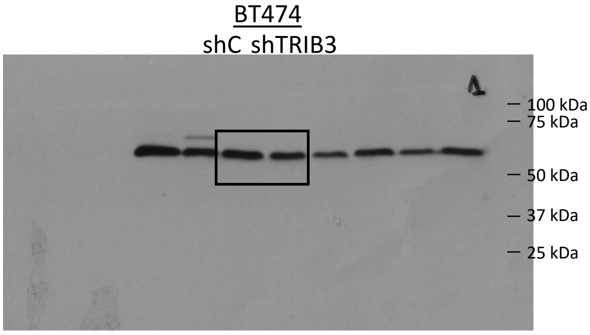

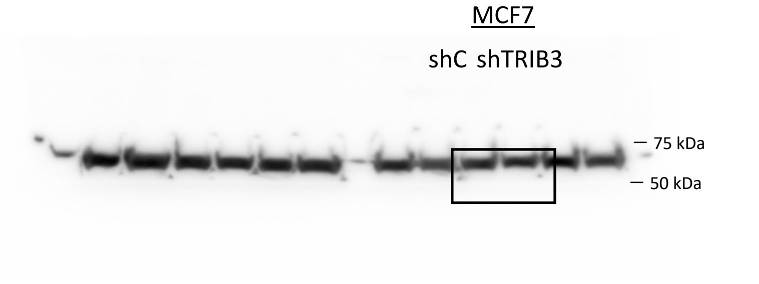

Fig. 2E

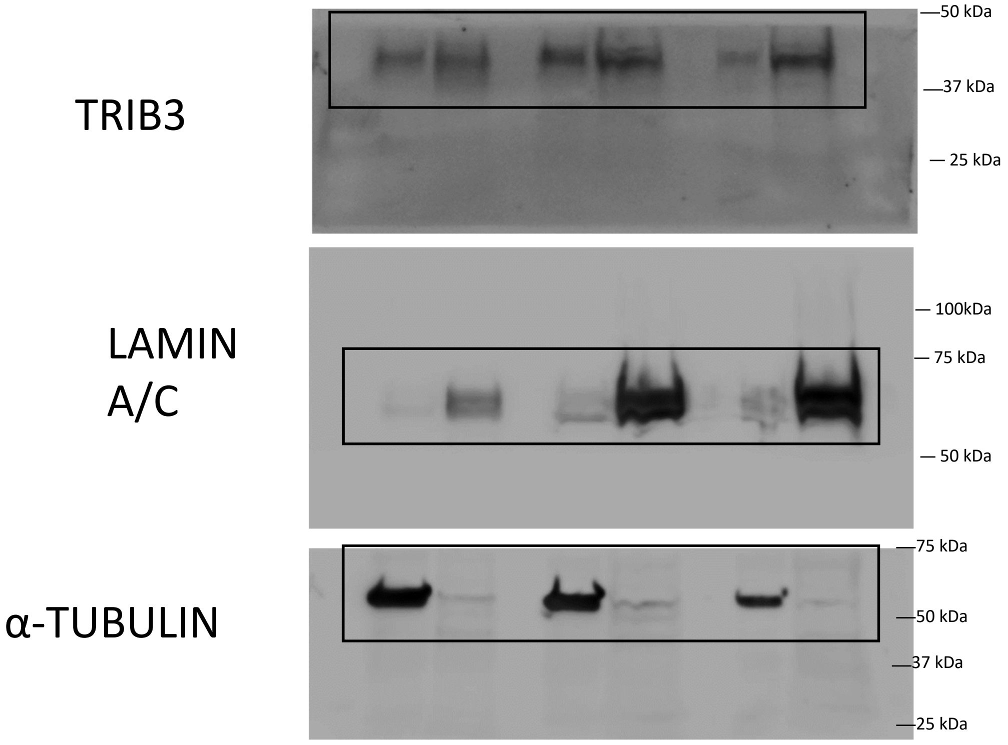
TRIB3
50 kDa
37 kDa
25 kDa
LAMIN A/C
100kDa
75 kDa
50 kDa
α-TUBULIN
75 kDa
50 kDa
37 kDa
25 kDa

Fig. 2F

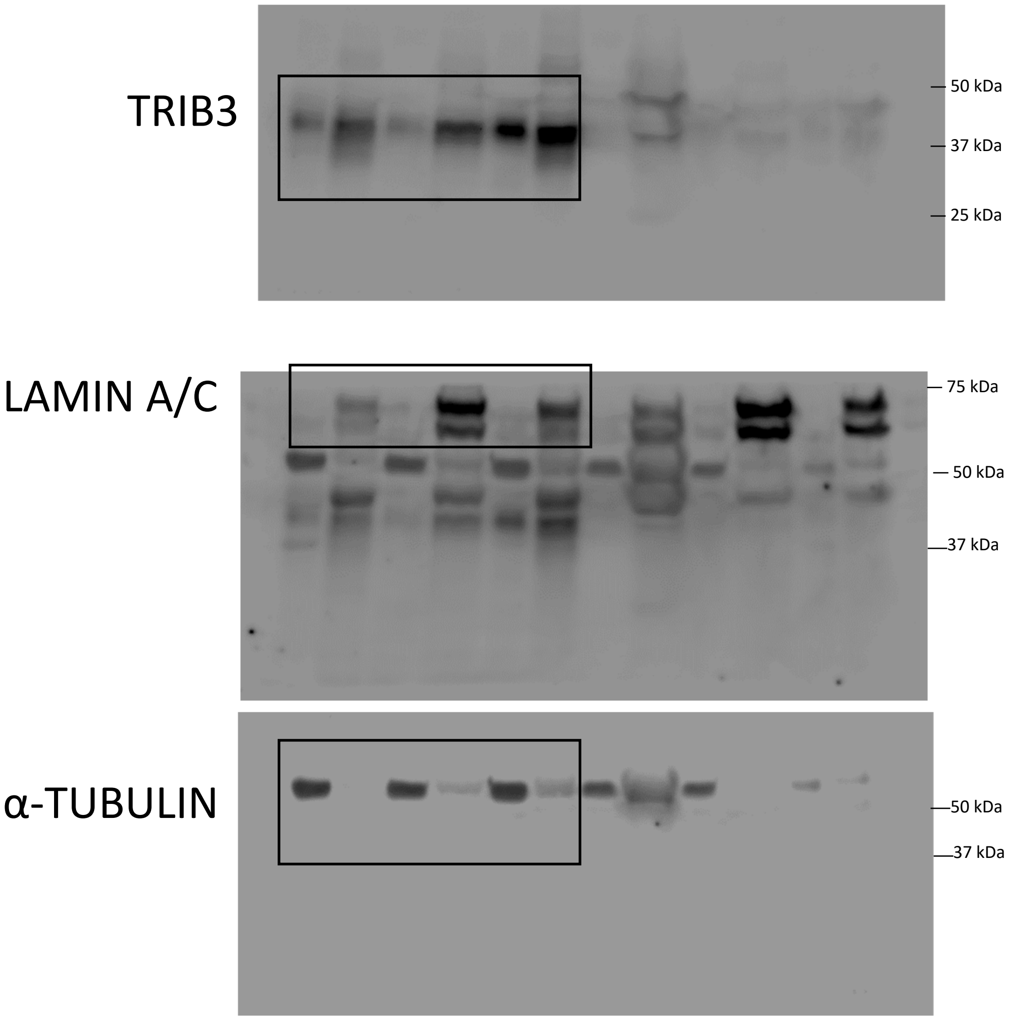
TRIB3
50 kDa
37 kDa
25 kDa
LAMIN A/C
75 kDa
50 kDa
37 kDa
α-TUBULIN
50 kDa
37 kDa

Fig. 2G

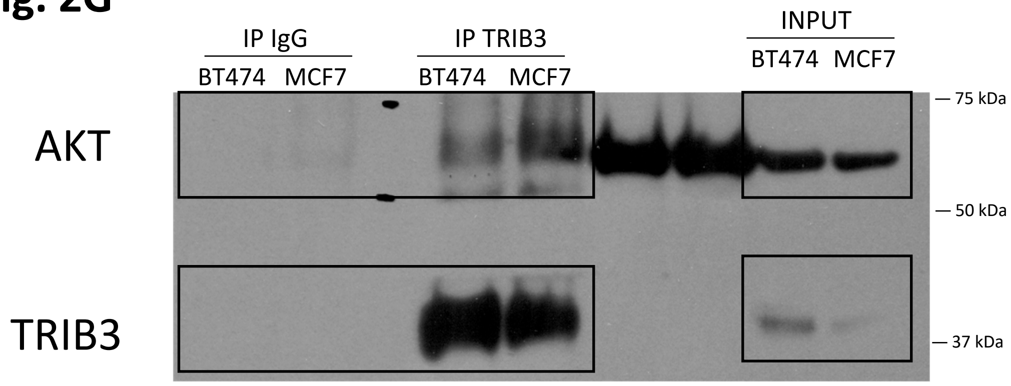
IP IgG
BT474 MCF7
IP TRIB3
BT474 MCF7
INPUT
BT474 MCF7
AKT
TRIB3
75 kDa
50 kDa
37 kDa

Fig. 2H

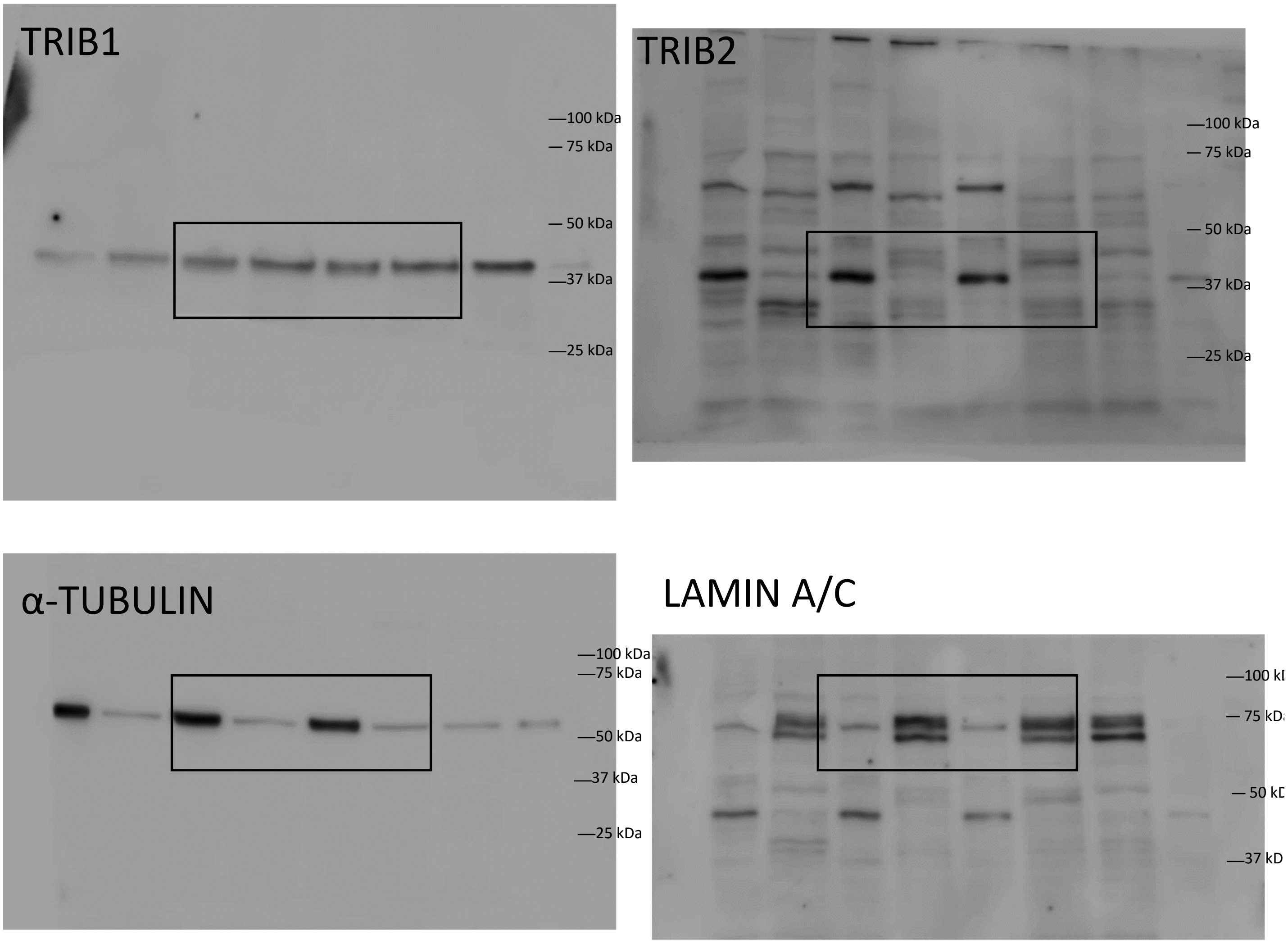
TRIB1
100 kDa
75 kDa
50 kDa
37 kDa
25 kDa
TRIB2
100 kDa
75 kDa
50 kDa
37 kDa
25 kDa
α-TUBULIN
100 kDa
75 kDa
50 kDa
37 kDa
25 kDa
LAMIN A/C

Fig. 4A

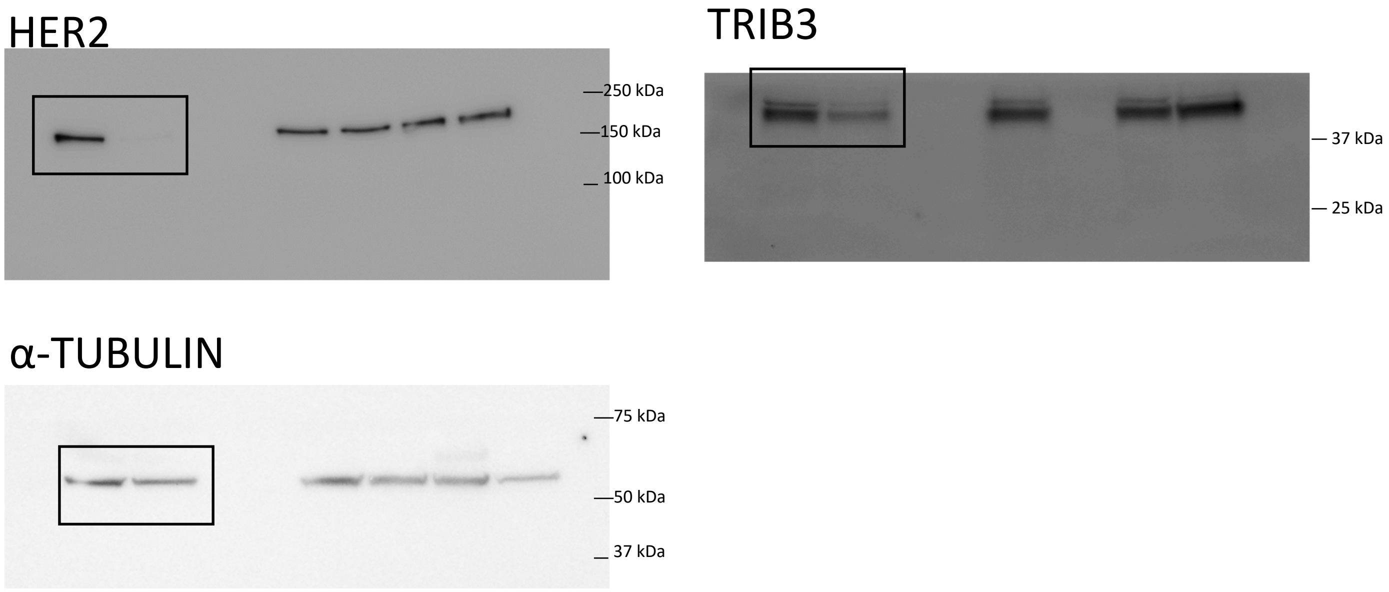
HER2
—250 kDa
—150 kDa
100 kDa
TRIB3
— 37 kDa
— 25 kDa
α-TUBULIN
—75 kDa
—50 kDa
37 kDa

Fig. 4B

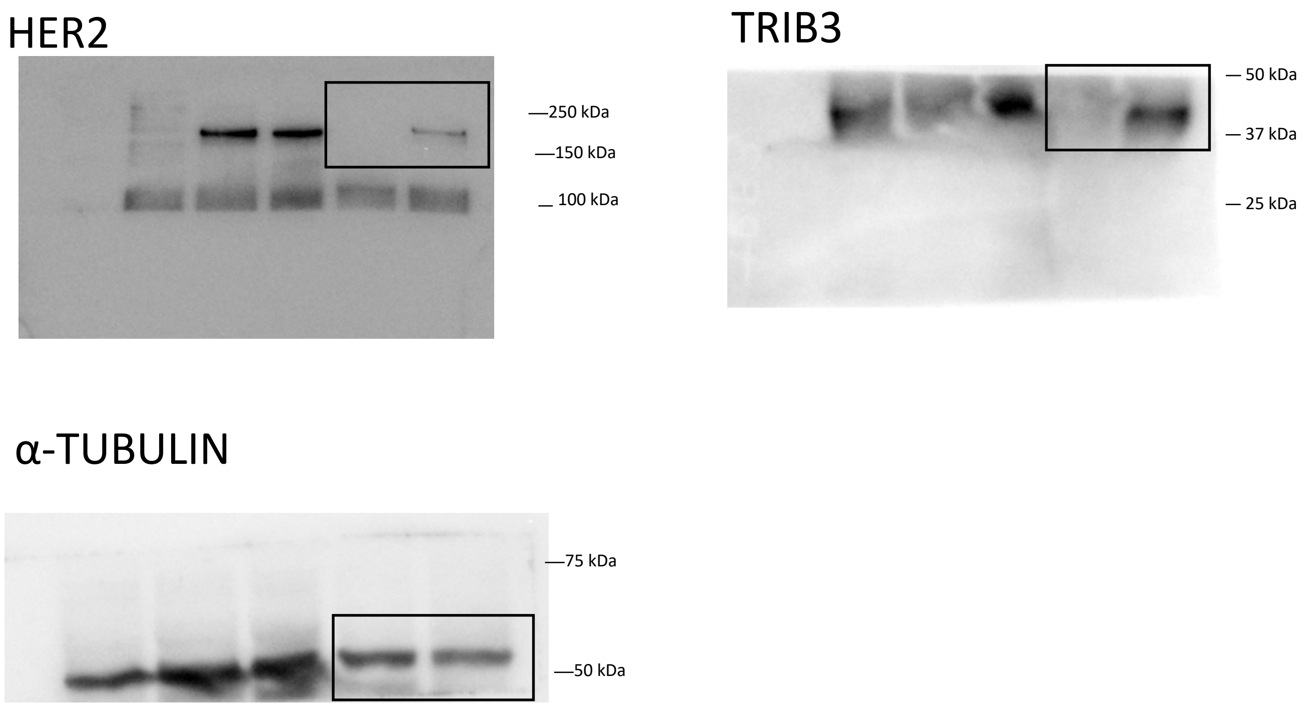
HER2
—250 kDa
—150 kDa
100 kDa
TRIB3
— 50 kDa
— 37 kDa
— 25 kDa
α-TUBULIN
—75 kDa
—50 kDa

Fig. 4D

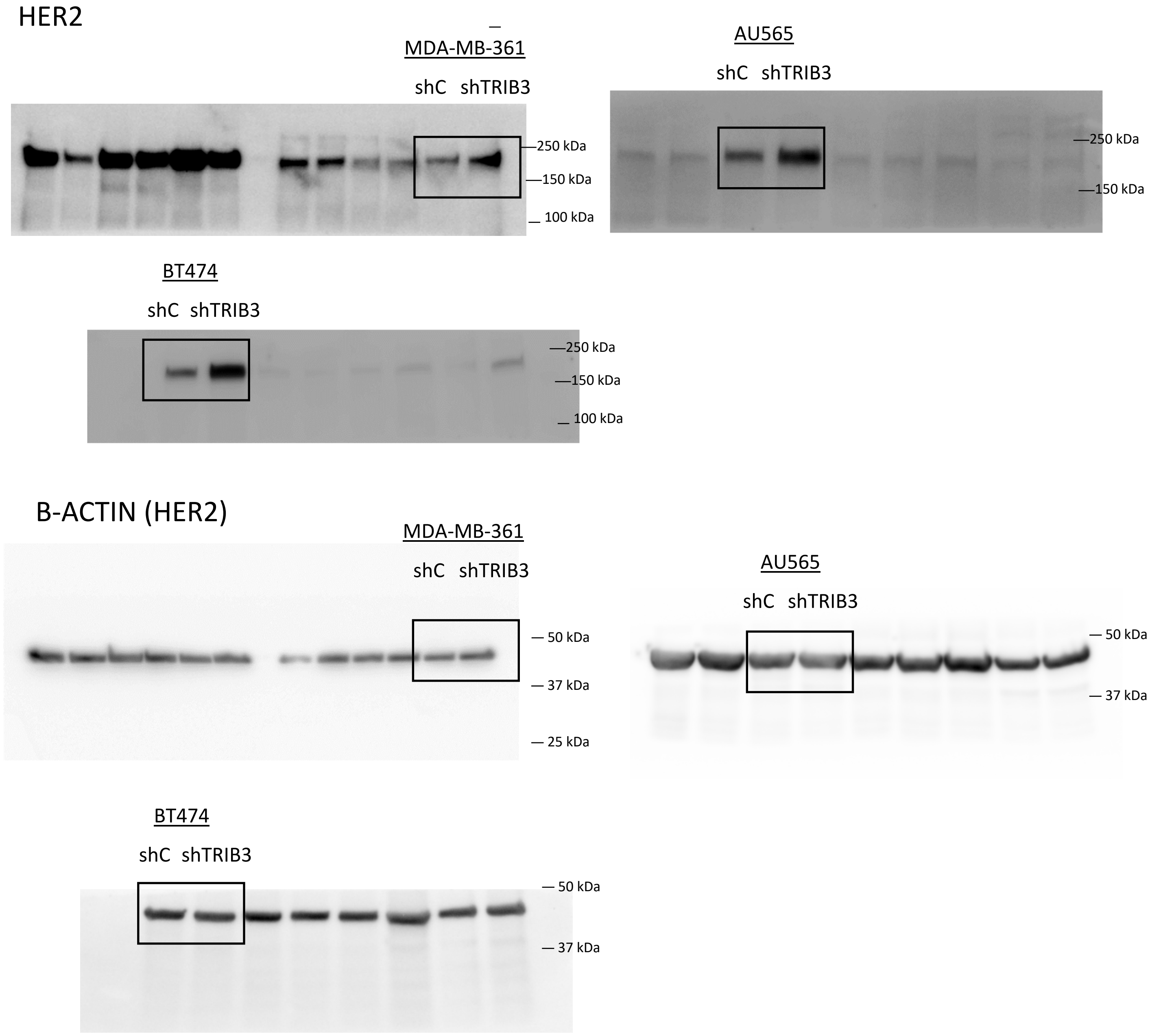
HER2
MDA-MB-361
shC shTRIB3
250 kDa
150 kDa
100 kDa
AU565
shC shTRIB3
250 kDa
150 kDa
BT474
shC shTRIB3
250 kDa
150 kDa
100 kDa
B-ACTIN (HER2)
MDA-MB-361
shC shTRIB3
50 kDa
37 kDa
25 kDa
AU565
shC shTRIB3
50 kDa
37 kDa
BT474
shC shTRIB3
50 kDa
37 kDa

Fig. 4E

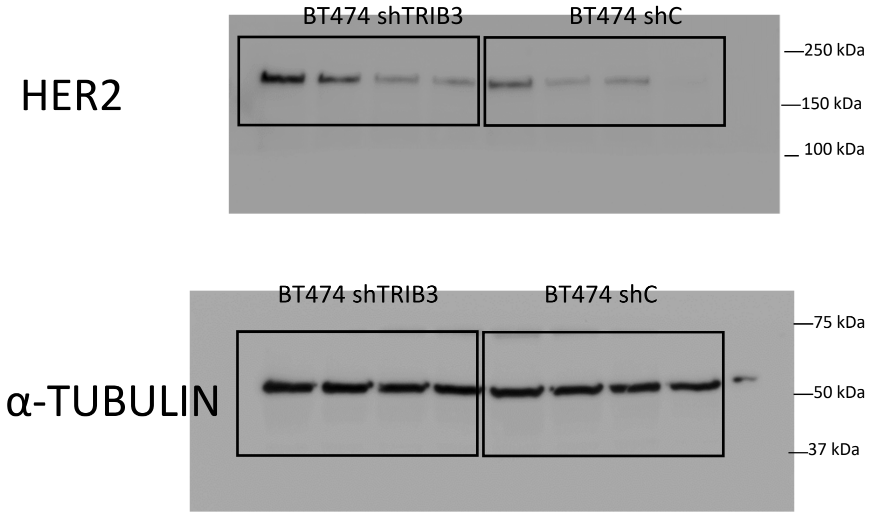
HER2
BT474 shTRIB3
BT474 shC
250 kDa
150 kDa
100 kDa
α-TUBULIN
BT474 shTRIB3
BT474 shC
75 kDa
50 kDa
37 kDa

Fig. S2A

TRIB3

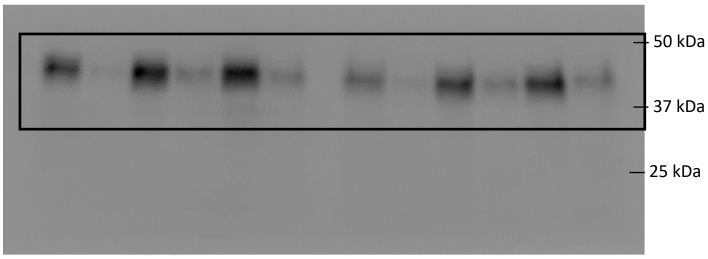

HSP90

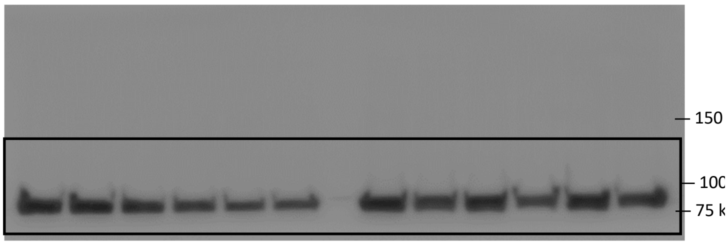

Fig. S2B

TRIB3

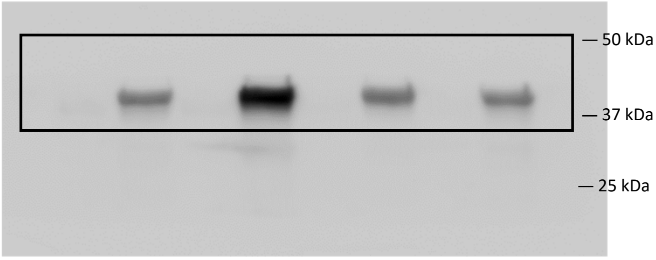

HSP90

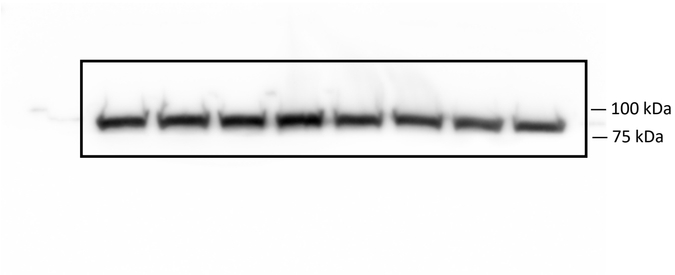

Fig. S3A

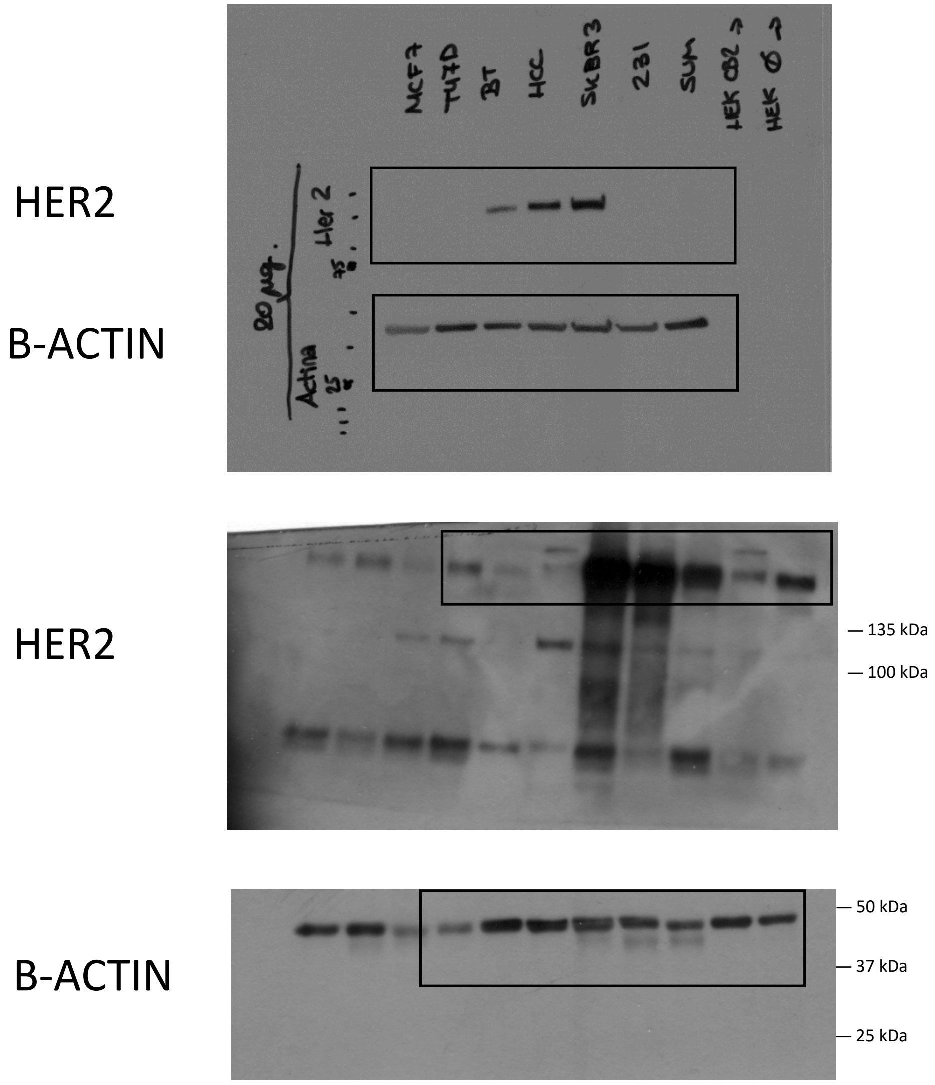
MCF7
T47D
BT
HCC
SKBR3
231
SUM
HER2
B-ACTIN
HER2
— 135 kDa
— 100 kDa
B-ACTIN
— 50 kDa
— 37 kDa
— 25 kDa

Fig. S3B

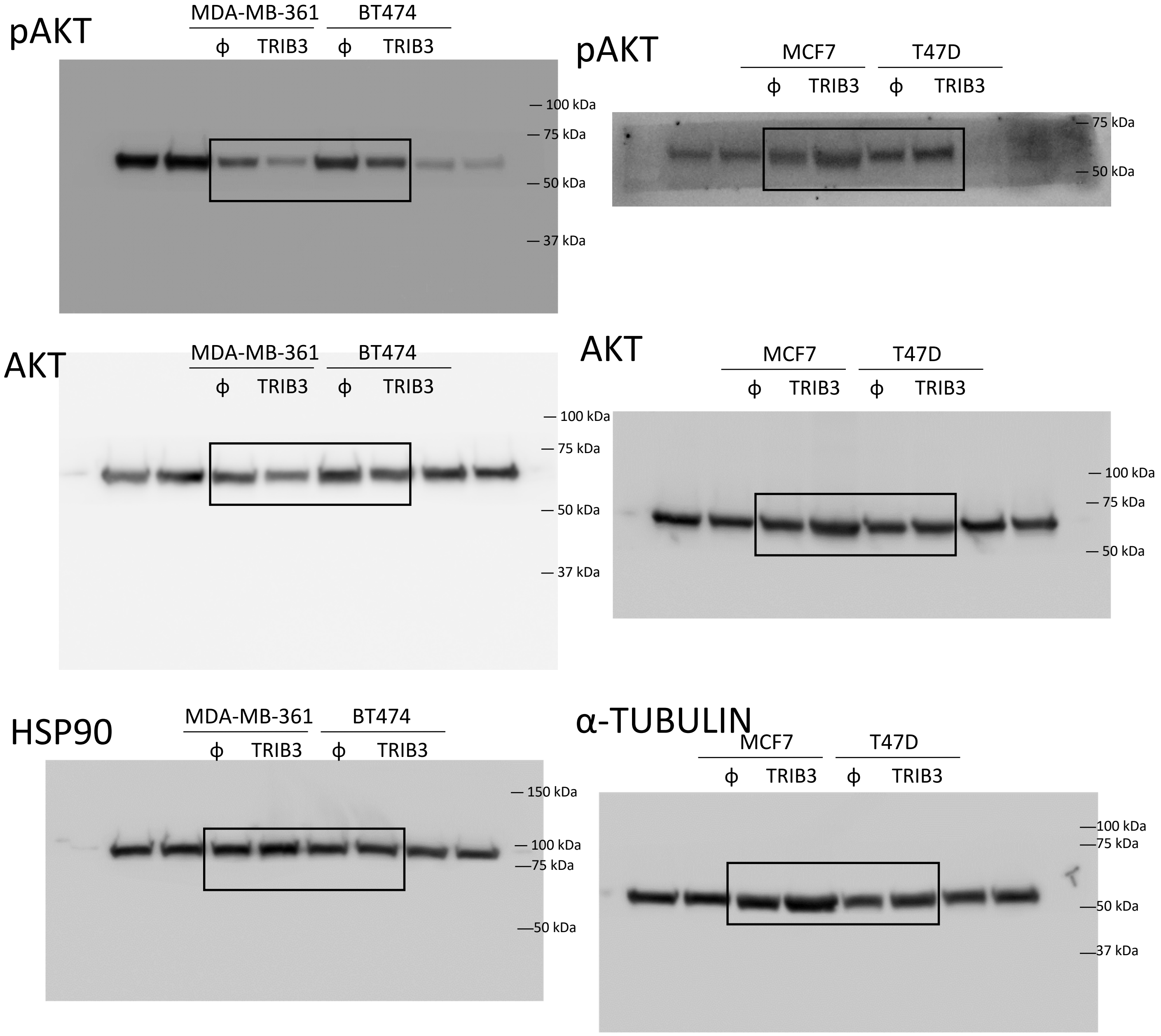
pAKT
MDA-MB-361
BT474
ϕ
TRIB3
ϕ
TRIB3
— 100 kDa
— 75 kDa
— 50 kDa
— 37 kDa
pAKT
MCF7
T47D
ϕ
TRIB3
ϕ
TRIB3
— 75 kDa
— 50 kDa
AKT
MDA-MB-361
BT474
ϕ
TRIB3
ϕ
TRIB3
— 100 kDa
— 75 kDa
— 50 kDa
— 37 kDa
AKT
MCF7
T47D
ϕ
TRIB3
ϕ
TRIB3
— 100 kDa
— 75 kDa
— 50 kDa
HSP90
MDA-MB-361
BT474
ϕ
TRIB3
ϕ
TRIB3
— 150 kDa
— 100 kDa
—75 kDa
—50 kDa
α-TUBULIN
MCF7
T47D
ϕ
TRIB3
ϕ
TRIB3
—100 kDa
—75 kDa
—50 kDa
—37 kDa

Fig. S3C

pAKT T308

MDA-MB-361

shC shTRIB3

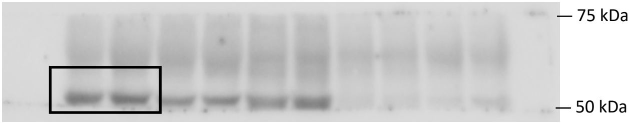

AU565

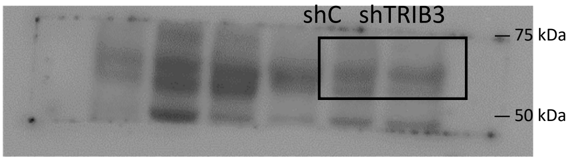

T47D

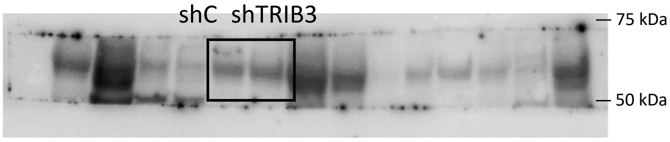

ZR75B

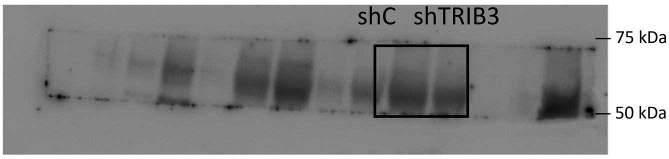

ACTIN

MDA-MB-361

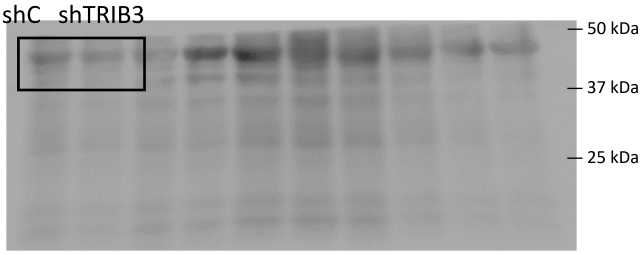

AU565

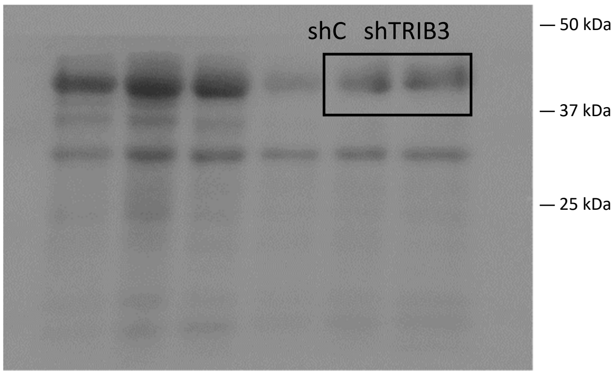

T47D

shC shTRIB3

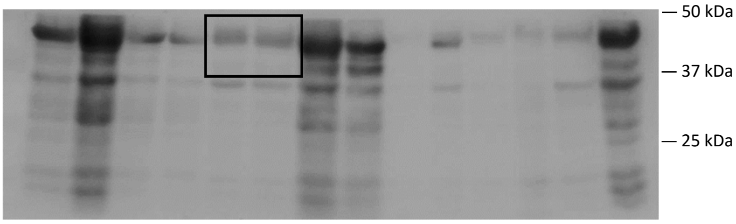

ZR75B

shC shTRIB3

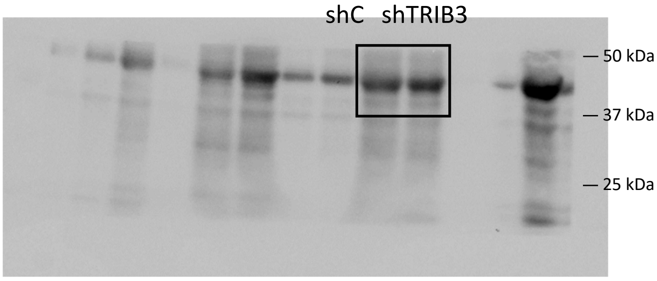

Supplement: Supplementary file 1 [file cancers-13-05307-s001.zip › cancers-1400452-supplementary.pdf]
